# Sampling with flows, diffusion, and autoregressive neural networks from a spin-glass perspective

**DOI:** 10.1073/pnas.2311810121

**Published:** 2024-06-24

**Authors:** Davide Ghio, Yatin Dandi, Florent Krzakala, Lenka Zdeborová

**Affiliations:** ^a^Information, Learning and Physics Laboratory, École Polytechnique Fédérale de Lausanne, Lausanne CH-1015, Switzerland; ^b^Statistical Physics of Computation Laboratory, École Polytechnique Fédérale de Lausanne, Lausanne CH-1015, Switzerland

**Keywords:** sampling, spin glasses, diffusion-generated models, flow-based models, autoregressive networks

## Abstract

Recent years witnessed the development of powerful generative models based on flows, diffusion, or autoregressive neural networks, achieving remarkable success in generating data from examples with applications in a broad range of areas. A theoretical analysis of the performance and understanding of the limitations of these methods remain, however, challenging. In this paper, we undertake a step in this direction by analyzing the efficiency of sampling by these methods on a class of problems with a known probability distribution and comparing it with the sampling performance of more traditional methods such as the Monte Carlo Markov chain and Langevin dynamics. We focus on a class of probability distribution widely studied in the statistical physics of disordered systems that relate to spin glasses, statistical inference, and constraint satisfaction problems. We leverage the fact that sampling via flow-based, diffusion-based, or autoregressive networks methods can be equivalently mapped to the analysis of a Bayes optimal denoising of a modified probability measure. Our findings demonstrate that these methods encounter difficulties in sampling stemming from the presence of a first-order phase transition along the algorithm’s denoising path. Our conclusions go both ways: We identify regions of parameters where these methods are unable to sample efficiently, while that is possible using standard Monte Carlo or Langevin approaches. We also identify regions where the opposite happens: standard approaches are inefficient while the discussed generative methods work well.

## Significance

Sampling from a given probability distribution is fundamental across various disciplines, including physics, signal processing, and artificial intelligence. In recent years, the ascendancy of flow, diffusion, and autoregressive neural network methods has led to remarkable achievements. The theoretical understanding of these methods is challenging. Here, we analyze the performance of these techniques and compare to more traditional methods like Langevin dynamics and Monte Carlo Markov chains, particularly in the context of spin glass models and related inference problems. Our findings underscore that these techniques fall short in sampling specific distributions at high temperatures, a task at which conventional Monte Carlo methods succeed. In high signal-to-noise ratio inference tasks, we pinpoint regions where these techniques surpass the traditional approaches.

The field of machine learning recently witnessed the development of powerful generative models able to produce new data-samples based on learning on datasets of existing samples. Among the most prominent ones achieving recent successes are flow-based models (1–5), diffusion-based models (6–9), and generative autoregressive neural networks (10–13). These approaches are achieving remarkable success in diverse areas such as image generation (14), language modeling (15), generation of molecules (16, 17), or theoretical physics (18). A theoretical understanding of the capabilities of these models, of their limitations and performance, remains, however, a challenge. A major aspect of these techniques is in the learning of the probability measure or its representation from examples. In this paper, we focus instead on a restricted setting where the probability distribution we aim to sample from is known beforehand. Our main goal is to contribute to setting the theoretical understanding of the capabilities and limitations of these powerful generative models.

When applied to parametric probability distributions, the generative models are indeed designed with the goal to provide samples uniform at random from the distribution. Here, we study whether they are able to efficiently sample from types of probability distributions that are encountered in the study of mean-field spin glasses and related statistical inference problems (19–24).

There are several benefits to studying this class of probability distributions. On the one hand, due to numerous studies in the statistical physics of disordered systems, we possess a comparatively good grasp of parameter regions where traditional sampling methods—like Monte Carlo sampling or Langevin dynamics—are effective and where they are not (25–30). On the other hand, the tools available for outlining the phase diagrams of these problems (20, 21, 31, 32) turn out to be highly effective in analytically describing the performance of generative techniques such as flow-based, diffusion-based, or autoregressive networks as samplers for the respective probability measures.

The above-mentioned tools, along with their mathematically rigorous counterparts, have recently been applied to the analysis and design of sampling algorithms in mean-field spin glass models in the context of stochastic localization (33–38). This was later found to have a close relationship with diffusion-based models (37, 38). In particular, El Alaoui et al. (36) and Montanari and Yu (37) showed how one can turn the message-passing denoising algorithms into samplers, a technique we shall use as well in the present study. While we could not find similar studies for flow-based models, closely related work on autoregressive networks exists on the decimation of message-passing algorithms used for finding solutions to the random K-satisfiability problem in refs. 39 and 40. Here, we build on these works and bring the following contributions:

- Using the formalism of stochastic interpolants (4, 41), we analyze sampling with flow-based methods, which leads to Bayesian denoising with an additive white Gaussian noise (AWGN). This turns out to be equivalent to what arises in the analysis of diffusion-based sampling and stochastic localization derived in refs. 36, 38, and 42.
- In the case of autoregressive generative networks, the analysis leads instead to a Bayesian denoising problem now correcting erased variables [the Binary Erasure Channel (43)] as in ref. 40.
- Focusing on prototypical exactly solvable models where one can perform asymptotic analysis, we then study the phase diagrams of the corresponding Bayesian denoising problems as a function of the strength of the signal-to-noise ratio.
- We investigate the feasibility of sampling using these techniques. We posit that these methods are capable of efficient sampling, provided there is no range in noise amplitude exhibiting the metastability characteristic of first-order phase transitions. If such metastability exists, the denoising becomes computationally hard.
- We locate these metastable regions in prototypical models, specifically the Ising and spherical *p*-spin models, the bicoloring of random hypergraphs problem, and a matrix estimation problem with a sparse spike.
- We also provide a GitHub repository with our numerical experiments in ref. 44; see https://github.com/IdePHICS/DiffSamp.

**Fig. 1.**
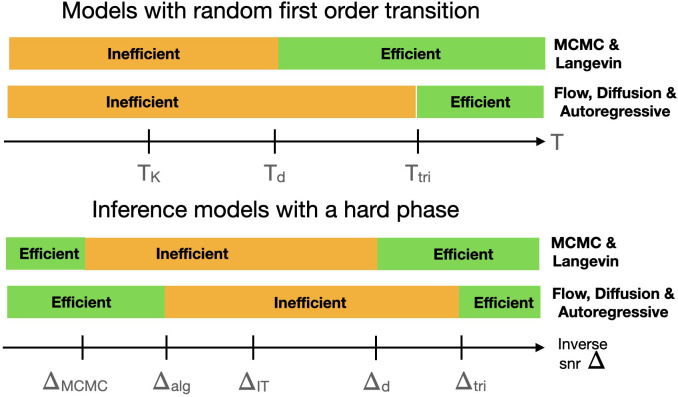
Schematic summary of the comparison of the efficiency of sampling with flow-based, diffusion-based, and autoregressive methods versus Langevin or Monte-Carlo approaches in spin glass models with a random first-order transition (*Top*), and in statistical inference models with a computationally hard phase (*Bottom*). The computationally hard phase in inference problems appears for $\Delta_{\mathrm{alg}}<\Delta <\Delta_{\mathrm{IT}}$ where efficient algorithms achieving close-to-optimal estimation error are not known and conjectured not to exist (45).

In terms of comparison between the flow-based, diffusion-based, or autoregressive generative models with more traditional Monte Carlo Markov chains (MCMC) and Langevin sampling procedures, the following picture emerges (summarized in Fig. 1) for two classes of models.

- Disordered models that exhibit a phase diagram of the random-first-order-theory (RFOT) type, also often called discontinuous one-step replica symmetry breaking (19, 46), are typical in the mean-field theory of glass transition (28), but they also appear in a variety of random constraint satisfaction problems (22). For such models, there exists a so-called dynamical temperature $T_{d}$ such that Monte Carlo Markov chains or Langevin algorithms are predicted to sample efficiently for $T>T_{d}$ (25–29) while the sampling problem is computationally hard for $T<T_{d}$. Recent work (47) showed empirically that the region $T<T_{d}$ is also hard when sampling with autoregressive neural networks. Our analysis of the currently used flow-based, diffusion-based, and autoregressive networks-based sampling procedures goes further and reveals analytically that they perform worse than the traditional techniques and that there is another temperature $T_{\mathrm{tri}}$, that depends on the detail of the method, such that for $T_{d}<T<T_{\mathrm{tri}}$ these generative methods do not sample efficiently while traditional procedures do.
- A second class of problems are the noisy random statistical inference problems with a hard phase and a statistical-to-computational gap (23, 24, 45, 48). In such problems, a good statistical inference can only be performed below a critical value of the noise (or inverse signal-to-noise ratio) $\Delta_{\mathrm{IT}}$, at least with infinite computational power. It turns out, however, that the best algorithms we know for such problems (in particular message-passing ones) are only able to work below a second threshold $\Delta_{\mathrm{alg}}$ and fail to learn in the “hard” region $\Delta_{\mathrm{alg}}<\Delta <\Delta_{\mathrm{IT}}$. The existing literature predicts an even more significant gap to exist in those cases for MCMC or Langevin-based samplers (30, 49, 50) down to a noise amplitude $\Delta_{\mathrm{MCMC}}<\Delta_{\mathrm{alg}}$. In this setting, however, it appears that flow-based, diffusion-based, and autoregressive network-based methods outperform the standard approaches and sample efficiently as soon as $\Delta <\Delta_{\mathrm{alg}}$.

## Further Related Works.

The present study of sampling algorithms owes a lot to recent lines of work. In particular, we shall follow the helpful approach of continuous-time flows based on stochastic interpolants (4, 41) as the starting point. Additionally, our way of setting up the statistical physics equivalent problem for a continuous-time flow method closely follows the one pioneered recently in refs. 36 and 42 for stochastic localization and generalized in ref. 37 for diffusion models.

The difficulty in denoising we uncover turns out to be connected to an easy-hard transition arising in statistical inference problems (23) often called the statistical-to-computational gap (24, 48). For these models, mean-field algorithms from spin glass theory, such as the Belief-Propagation (BP) equations (21) and the Approximate-Message-Passing (AMP) (31), or the equivalent Thouless–Anderson–Palmer (TAP) (51) approach, are believed to be among the most efficient one (52). Using AMP for sampling via the diffusion method (or via stochastic localization) was discussed recently in refs. 36 and 37. The resulting algorithm turns out to have very close connections to the so-called reinforcement BP (53), and a direct connection exists between the analysis in the present paper and the reinforced and decimated version of message-passing algorithms (39, 54–56).

Finally, we note that since we assume that the model is known, we are not discussing here the limitations of training denoisers models from a limited number of data points. This difficulty was discussed recently in ref. 57 in the context of statistical physics models.

## Sampling with Flow and Diffusion-Based Models

We start by discussing continuous-time flow-based models (3–5). The goal is to sample a vector $x_{0}\in R^{N}$ from a so-called target distribution $P_{0}\left(x_{0}\right)$. Continuous-time flow-based models (3–5, 58) achieve this by constructing an ODE (flow) that continuously transforms Gaussian noise $z∼N(0,I_{N})$ to a sample $x_{0}∼P_{0}$. One way to obtain such a flow is by inverting the time-evolving law of stochastic or deterministic processes bridging from a sample $x_{0}∼P_{0}$ to a Gaussian noise $z∼N(0,I_{N})$. Such a continuous-time reverse process underlies a wide range of flow-models (4, 5) as well as the score-based diffusion models (9). In practice, we would only observe samples from the distribution $P_{0}\left(x_{0}\right)$, whereas in this paper, we aim to focus on the limitations of these procedures, we will thus assume that the distribution $P_{0}\left(x_{0}\right)$ is known as this can only render the task easier than when only samples from $P_{0}\left(x_{0}\right)$ are available.

To present this method clearly, we find it convenient to use the formalism of stochastic linear interpolants introduced in refs. 4 and 41. Starting from a vector $x_{0}$ sampled from $P_{0}$ and a Gaussian vector z, we consider the process—or one-sided stochastic interpolant—defined by

[1] $$y(t)=\alpha (t)x_{0}+\beta (t)z,\;\;\;\;\;\;\;\;\;t\in [0,1],$$

where the functions α and β are generic, but constrained by the following relations:

[2] $$\begin{array}{c} \alpha (0)=\beta (1)=1;\;\alpha (1)=\beta (0)=0;\; \end{array}$$

[3] $$\begin{array}{c} \forall t\in [0,1]:\alpha (t)\geq 0,\;\;\overset{\cdot }{\alpha }(t)\leq 0,\;\;\beta (t)\geq 0,\;\overset{\cdot }{\beta }(t)\geq 0 \end{array}$$

such that indeed we have $y\left(0\right)=x_{0}$ and y(1)=z. Suppose that $P_{0}\left(x_{0}\right)$ admits a density $\rho_{0}$ w.r.t the Lebesgue measure. As shown in (41), see also *SI Appendix*, section 5, that the probability density ρ(y(t)) associated to the measure $P_{t}$ of the random variable y(t) satisfies the following transport equation:

[4] $$\begin{array}{c} \partial_{t}\rho \left(y,t\right)+\nabla \cdot \left(b\left(y,t\right)\rho \left(y,t\right)\right)=0, \end{array}$$

where we defined the velocity field

[5] $$\begin{array}{c} b\left(y,t\right)=E\left[\partial_{t}y\left(t\right)|y\left(t\right)=y\right]=E\left[\dot{\alpha }\left(t\right)x_{0}+\dot{\beta }\left(t\right)z|y\left(t\right)=y\right]. \end{array}$$

Indeed, b(y,t) is simply the expected velocity of y(t) conditioned on being at y at time t. In *SI Appendix*, section 5, we also provide a formal definition of Eq. **5** based on the analysis in ref. 4, along with a discussion of the case when the measure $P_{0}\left(x_{0}\right)$ is discrete.

Eq. **1** defines a forward process interpolating from $x_{0}$ to z. Eq. **4** further reveals that, in law, y(t) can be obtained by applying the vector field (Eq. **5**) starting from $x_{0}$ at time t=0. The algorithm proposed by Albergo and Vanden-Eijnden (4) relies on applying the velocity field in the reverse direction of time. Concretely, it relies on approximating the unique solution to the following ordinary differential equation (ODE) starting from a random Gaussian initial condition from t=1, back to t=0:

[6] $$\begin{array}{c} \frac{dY(t)}{\mathrm{dt}}=b\left(Y\left(t\right),t\right). \end{array}$$

Eq. **4** then implies that the random variable defined by Eq. **6** solved backward in time from the final value $Y_{t=1}=z∼N\left(0,I_{N}\right)$ is also distributed according to ρ(y(t)) (which is nothing but the continuity equation for the flow defined by Eq. **6**) and, in particular, is distributed as the desired target $P_{0}\left(x_{0}\right)$ at time t=0. If we can numerically solve this ODE, then we have a sampling algorithm for $P_{0}\left(x\right)$.

In order to do that, we discretize Eq. **6** using the forward Euler method (in reverse time) to write

[7] $$\begin{array}{c} Y_{t-\delta }=Y_{t}-\delta \;b\left(Y_{t},t\right). \end{array}$$

Noticing that we can rewrite the vector field using Eq. **1** as

[8] $$\begin{array}{cc} b(Y_{t},t) & =E\left[\overset{\cdot }{\alpha }(t)x_{0}+\frac{\overset{\cdot }{\beta }(t)}{\beta (t)}\beta (t)z|y(t)=Y_{t}\right]\\ & =E\left[\overset{\cdot }{\alpha }(t)x_{0}+\frac{\overset{\cdot }{\beta }(t)}{\beta (t)}\left(y(t)-\alpha (t)x_{0}\right)|y(t)=Y_{t}\right]\\ & =\frac{\overset{\cdot }{\beta }(t)}{\beta (t)}Y_{t}+\left(\overset{\cdot }{\alpha }(t)-\frac{\beta (t)\alpha (t)}{\overset{\cdot }{\beta }(t)}\right)E\left[x_{0}|y(t)=Y_{t}\right], \end{array}$$

we put back Eq. **8** in Eq. **7** to reach

[9] $$\begin{array}{c} Y_{t-\delta }\;=\;\left(\text{​}1-\delta \frac{\overset{\cdot }{\beta }(t)}{\beta (t)}\text{​}\right)\text{​}Y_{t}-\delta \left(\text{​}\overset{\cdot }{\alpha }(t)-\frac{\overset{\cdot }{\beta }(t)\alpha (t)}{\beta (t)}\text{​}\right)E\left[x_{0}|y(t)=Y_{t}\right] \end{array}$$

which, given the initial condition $Y_{t=1}=z$, will evolve back to a sample from the target $Y_{t=0}∼P_{0}$, provided that we are able to estimate $E\left[x_{0}|y\left(t\right)=Y_{t}\right]$ well at each time t. In Algorithm 1, we report a schematic implementation of the resulting flow-based sampling technique. In *SI Appendix*, section 5, we further discuss the effect of discretization and the associated sampling guarantees under access to a perfect denoiser.

### Diffusion-Based Models and SDEs.

The flow-based algorithm (Algorithm 1) from ref. 4 relies on replicating the law of the interpolant y(t) defined by Eq. **1** through the ODE defined by Eq. **6**. Interestingly, however, the same law for y(t) can be obtained via a stochastic differential equation (SDE). Indeed, when considering a forward diffusion process applied to the data distribution, the same law for y(t) can be retrieved through an associated time-reversed SDE (or ODE) (9, 59). It turns out that both flow-based (ODE) methods and diffusion-based (SDE) ones associated with the above processes [as well as stochastic localization (33–35)] require estimating the posterior-mean $E\left[x_{0}|y\left(t\right)=Y_{t}\right]$, i.e., averages w.r.t a “tilted” probability measure. Utilization of such an evolving tilted measure for sampling was done first in the stochastic-localization-based sampling algorithm in refs. 36 and 37 and related to generic diffusion models in ref. 38. The reliance of both the ODE and SDE-based approaches on the same tilted measure is a consequence of the posterior mean (or equivalently the score) yielding both the velocity field for the ODE defined by Eq. **6** and the drift term for the equivalent SDE as noted in refs. 4, 9, and 41. Since the law of y(t) remains the same with the ODE or SDE-based approaches, the deterministic evolution of the tilted measure $P(x|y\left(t\right)=Y_{t})$ matches in law with that of the associated stochastic localization process (38). This is convenient for our analysis since this means that all the phase transitions discussed in our work apply to flow-based methods, diffusion-based ones, and stochastic localization approaches.


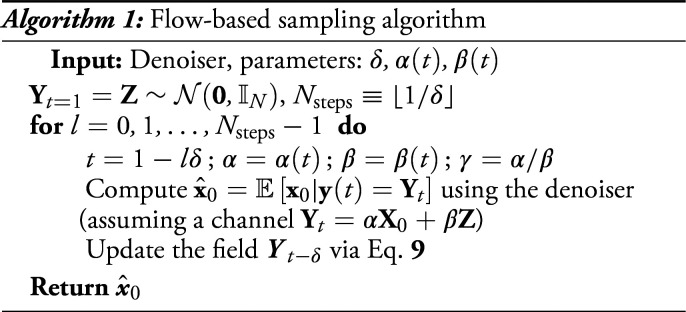


### The Posterior Average and Bayes-Optimal Denoising.

The key difficulty is thus to estimate the average $E\left[x_{0}|y\left(t\right)=Y_{t}\right]$. This is nothing but the Bayes-optimal denoising where $Y_{t}$ is a noisy observation of $x_{0}$ corrupted by additive white Gaussian noise in the form of Eq. **1**.

The Bayes-optimal denoising estimator $\hat{x}$ (optimal in the sense of minimizing the mean-squared-error between the estimator and $x_{0}$) is to use the posterior mean $\hat{x}=E\left[x_{0}|y\left(t\right)=Y_{t}\right]$ (60). Determining this average can be computationally hard, as it involves an integral in high-dimension. In practical machine learning setups, one learns the denoiser using a neural network trained on data coming from the distribution. Thus, the main narrative of the diffusion and flow-based methods is that if one can access to a good denoiser, then one can turn it into a sampler.

In this paper, we aim to focus on the limitations of this procedure, and we shall hence assume that the task of obtaining a denoiser has been completed to near perfection by focusing on target distributions $P_{0}\left(x_{0}\right)$ stemming from problems studied in statistical physics for which the Bayes-optimal denoising problem including its algorithmic feasibility has been extensively investigated. Before turning to these models, we write the denoising problem in terms that are more familiar in the statistical physics but also in the information-theoretic context.

Let us consider again the process defined by Eq. **1**. Using the Bayes theorem, we can see that the posterior distribution of the sample conditioned on the observation $y\left(t\right)=Y_{t}$ is given by

[10] $$\begin{array}{cc} P(x|y(t) & =Y_{t})=\frac{P_{0}\left(x\right)P\left(y\left(t\right)=Y_{t}|x\right)}{P(y\left(t\right)=Y_{t})}\\ & =\frac{1}{Z(Y_{t})}\exp\left(\frac{\alpha (t)}{\beta {\left(t\right)}^{2}}\left\langle Y_{t},x\right\rangle -\frac{\alpha {\left(t\right)}^{2}}{2\beta {\left(t\right)}^{2}}{\left||x|\right|}^{2}\right)P_{0}\left(x\right), \end{array}$$

where in the last line, we put all the terms not depending on x inside the normalization aka partition function Z.

We now recall that the law of $Y_{t}$ can be obtained through an observation of a sample from the target distribution $x_{0}∼P_{0}$ through the AWGN channel rescaled by the factors α(t) and β(t) as in Eq. **1**.

Here, we denote $x_{0}$ as the “truth” signal and make the difference between the dummy variable x in the integral. We can hence rewrite the measure in Eq. **10** further as

[11] $$\begin{array}{cc} P_{\gamma }\left(x|x_{0},z\right) & \propto e^{\gamma {\left(t\right)}^{2}\left\langle x,x_{0}\right\rangle +\gamma \left(t\right)\left\langle z,x\right\rangle -\frac{\gamma {\left(t\right)}^{2}}{2}{\left\|x\|\right|}^{2}}P_{0}\left(x\right), \end{array}$$

where $z∼N(0,I_{N})$ and where we have defined the effective signal-to-noise ratio

[12] $$\begin{array}{c} \gamma \left(t\right)=\frac{\alpha (t)}{\beta (t)}. \end{array}$$

We see that, by construction, γ will be such that γ(1)=0 and γ(0)=+∞. We will refer to $P_{\gamma }$ as the tilted measure, and it will be a central object in the remaining discussion. We have added the index γ in the notation of the distribution $P_{\gamma }$ to distinguish it from other considered probability distributions from now on.

## Autoregressive Networks and Ancestral Sampling

Let us now discuss the second type of sampling algorithm considered in this paper—the autoregressive networks. A classical way of sampling a vector $x\in R^{N}$ from a target distribution $P_{0}\left(x\right)$ in computer science and statistics is sequential sampling or ancestral sampling: one starts by computing the marginal probability of the first coordinate $P_{0}\left(x_{1}\right)$, and samples $x_{1}$ accordingly. Subsequently, the algorithm generates all the coordinates for a sample, by sampling each coordinate conditioned on its “parent” coordinates (61). In the simplest case, the distribution of each coordinate is assumed to depend on all previous coordinates. Thus, after sampling $x_{1}$, for each subsequent node, one looks at the distribution $P_{0}\left(x|x_{1}\right)$, one considers the marginal distribution of $x_{2}$, etc. Of course, marginalization of a high-dimensional probability distribution is in general hard, and the strategy used in autoregressive networks (62) is to directly learn from data a probability distribution written in the (autoregressive) form $P_{0}\left(x\right)=P_{0}\left(x_{1}\right)P_{0}\left(x_{2}|x_{1}\right)\cdots P_{0}\left(x_{N}|x_{1},\cdots ,x_{N-1}\right)$ with each term being represented via a neural network. While this decomposition works for any ordering of the components, in practical applications the order may be relevant (this is an important point in ancestral sampling). In the present paper, we will consider the order to be random.

We showed in the previous section that sampling through diffusion in our formalism boils down to the performance of a sequence of denoising problems, where in information theory terms the signal is observed through an additive white Gaussian noise channel. Analogously to that, we can interpret the autoregressive networks, or its ideal version sequential sampling, as the estimation of the marginal when a fraction θ of entries of one configuration sampled uniformly at random from the target $P_{0}$ is revealed exactly. To come back to the sampling scheme, reversing this process is equivalent to fixing one variable at a time from the marginal conditioned to the variables previously fixed until all variables are fixed. In statistical physics, this procedure has been studied and analyzed under the name decimation (40). In Algorithm 2, we report a schematic implementation of the autoregressive-based sampling technique.

In information-theoretic terms, this corresponds to a denoising problem under the so-called binary erasure channel (BEC) in which a transmitter sends a bit, and the receiver either receives the bit correctly or with some probability 1−θ receives a message that the bit was not received but “erased” instead.

Repeating the way of thinking we used for sampling with diffusion, we now want to analyze the denoising for such models and write the modified measure

[13] $$\begin{array}{c} P_{\theta }\left(x|x_{0},S_{\theta }\right)\propto P_{0}\left(x\right)\prod_{i\in S_{\theta }}\delta (\left[x_{i}-{\left[x_{0}\right]}_{i}\right), \end{array}$$

where $S_{\theta }$ is the set of revealed variables of size θN. We can also think of these variables as pinned to their ground truth value $x_{0}$ and consequently call the measure $P_{\theta }\left(x|x_{0},S_{\theta }\right)$ the pinning measure. From a statistical physics point of view, the new measure can again be thought of as the original one, but with an “infinite” magnetic field pointing to the direction of the components of $x_{0}$ for an expected fraction θ of components. We are again interested in the marginals of $P_{\theta }$ that we will denote $\hat{x}\left(\theta \right)$. The evolution of the pinning measure can also be interpreted as a coordinate-by-coordinate stochastic localization process (35).


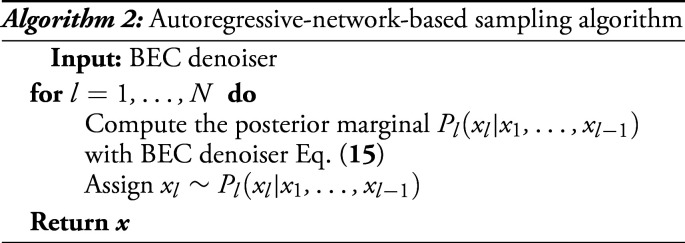


## Properties of Bayesian-Optimal Denoising

In both the cases—diffusion and autoregressive—we see that one has to perform an optimal denoising based on an observation y. In the case of diffusion, we have access to an observable where a sample from the target $x_{0}∼P_{0}$ is polluted by an AWGN channel:

[14] $$\begin{array}{c} y=\alpha x_{0}+\beta z, \end{array}$$

with $z∼N(0,I_{N})$, while for autoregressive, it is polluted by the BEC channel where for every i=1,⋯,N independently

[15] $$\begin{array}{c} y_{i}=\left\{\begin{array}{cc} {\left[x_{0}\right]}_{i} & \;\text{with probability}\;\;\theta \\ {}* & \;\text{otherwise} \end{array}\right.. \end{array}$$

Our goal is now to study the properties of such channels and the corresponding Bayes-optimal denoisers. A crucial point is that these Bayes-optimal estimation problems lead to the Nishimori identities and the single state (or replica symmetric in the spin glass jargon) properties of the measures $P_{\gamma }$, Eq. **11**, and $P_{\theta }$, Eq. **13**; see, e.g., the review (23). While these are classical properties, we remind their derivations in *SI Appendix* and state informally their form and main consequences here.

Concretely, we shall be interested in the evolution in time [or equivalently in γ∈[0,∞[ (AWGN) and θ∈[0,1] (BEC)] of the following order parameters, or overlaps:

[16] $$\mu (\gamma )\equiv \frac{1}{N}E[\hat{x}(\gamma )\cdot x_{0}],$$

[17] $$\chi (\gamma )\equiv \frac{1}{N}E[||\hat{x}(\gamma )|{|}^{2}].$$

The same definitions hold if we replace γ by θ, i.e., for the autoregressive process instead of the diffusive. The expectations are over the disorder $x_{0}$ and z. The single state property relates to the self-averaging of these order parameters: Almost anywhere in γ (AWGN) or θ (BEC), the equilibrium measure $P_{\gamma }$ and $P_{\theta }$ is a single “phase” probability measure, where overlap and order parameter are self-averaging, i.e., concentrate to a single scalar quantity as N→∞. Note that the “almost anywhere” is not a void statement: for instance, if one is sampling a complicated glassy problem such as, say, the Sherrington–Kirkpatrick model (20), strictly at γ=0, the Boltzmann measure is complex and overlaps are not self-averaging. For almost all values of γ (that is, except at some critical threshold values), the measure then corresponds to a much simpler single-phase problem, and in particular, there is no phenomenon akin to a static glassy transition or replica-symmetry-breaking; thus, the replica symmetric assumption is sufficient to describe the thermodynamic behavior of these measures. This will be instrumental in the exact asymptotic analysis.

While such results have a long history (63, 64), their proof in the present context follows from the analysis of optimal Bayesian denoising, and in particular the I-MMSE theorem and the fluctuation–dissipation one (32, 65–67), for the AWGN channel, and by the study of the so-called pinning lemma (68, 69) in the BEC channel, that also has roots in the study of the glass transition (29, 70).

A second property, often called the Nishimori symmetry in physics (23, 64), follows from Bayes theorem and states that for optimal Bayesian denoising one has μ(γ)=χ(γ) in the diffusion setting and μ(θ)=χ(θ) in the autoregressive one. We defer the details to *SI Appendix*, section 4.

Note that while we cannot experimentally compute μ(γ) in simulations, we can instead compute numerically χ(γ) which is just the norm of the estimator. The study of phase transitions in such problems is thus reduced to the behavior of a single scalar quantity.

## Prototypical Exactly Analyzable Models

We will now analyze the properties of the tilted measure $P_{\gamma }$ and the pinning measure $P_{\theta }$ for several concrete cases of the target measure $P_{0}$. This will be possible exactly in the thermodynamic limit N→∞. We shall focus on several classical problems from spin glass theory, statistical inference, and constraint optimization problems, but analogous analysis of the tilted and pinning measures can be done for many other problems for which the phase diagram was obtained via the replica or the cavity method (20, 21).

First, we shall start by studying models that present a so-called random first-order transition (28, 71), or in replica parlance, a discontinuous one-step replica symmetry breaking phenomenology (20). There are two crucial temperatures in RFOT systems. The so-called Kauzmann temperature $T_{K}$ below which the system behaves as an ideal glass and the so-called dynamical temperature $T_{d}$ that is defined as the temperature at which the point-to-set correlation length diverges and consequently Monte Carlo or Langevin dynamics equilibration time diverges as well (28, 29). It is a widely accepted conjecture that no efficient method can sample such models in their low temperature $T<T_{d}$ glassy phase [and in fact, one can prove such hardness for classes of algorithms (36, 72)]. We shall focus on the “paramagnetic” phases of these models $T>T_{d}$ for which the Monte Carlo or Langevin equilibration time is known to be finite and hence efficient sampling with MCMC or Langevin is possible. Specifically, we shall consider the following models:

- **The Ising and spherical p-spin glass** (19), whose Hamiltonian reads (for p=3):
  [18] $$\begin{array}{c} H\left(x\right)=-\frac{\sqrt{3}}{N}\sum_{i<j<k}J_{\mathrm{ijk}}x_{i}x_{j}x_{k}, \end{array}$$
  with $J_{\mathrm{ijk}}∼N\left(0,1\right)$. The Boltzmann distribution is then $P_{0}\left(x\right)\propto \exp\left(-\beta H\left(x\right)\right)$ with β=1/T. In the Ising case, we take $x_{i}=\pm 1$, for i=1,⋯,N, in the spherical case $x\in S^{N-1}$ (even though, since we are discussing the high-temperature phase, we shall use the equivalent Gaussian model). This is one of the most studied models in spin glass theory.
- **NAE-SAT or bicoloring**: Another class of popular models arises in the context of constraint satisfaction problems, e.g., random satisfiability problem or random graph coloring (22). Here, we shall focus on a prototypical case from this class, the problem of coloring random k-hypergraphs with two colors. This model was studied using statistical physics techniques in refs. 73–75. Numerous rigorous results for this model were also established, e.g., in refs. 76 and 77. The probability distribution is the following:
  [19] $$\begin{array}{c} P_{0}\left(x\right)\propto \prod_{a=1}^{M}\omega \left(x_{\partial a}\right),\;x\in {\left\{-1,+1\right\}}^{N}, \end{array}$$
  $$\begin{array}{c} \alpha =\frac{M}{N},\;\omega \left(x_{1},\cdots ,x_{k}\right)=\left\{\begin{array}{cc} 0 & \;\text{if}\;\sum_{i=1}^{k}x_{i}=\pm k\\ 1 & \;\text{otherwise} \end{array}\right., \end{array}$$
  where by $x_{\partial a}$, we refer to the group of k variables entering into the clause a. Again, this model presents a RFOT phenomenology, with a dynamical/clustering transition $\alpha_{d}$. The literature supports the property that MCMC is able to sample efficiently for $\alpha <\alpha_{d}$ and is not for $\alpha >\alpha_{d}$ (22, 29, 78).

In these models, by studying the tilted/pinning measures, we shall see the limitations of flow-based, diffusion, and autoregressive sampling methods that will fail at temperature $T_{d}<T<T_{\mathrm{tri}}$ and constraint density $\alpha_{\mathrm{tri}}<\alpha <\alpha_{d}$. These methods thus turn out to be not as performing as MCMC/Langevin approaches above the dynamical transition. We expect this to be the case for any model with RFOT phenomenology. Every cloud, however, has its silver lining, and we shall also note that there is a class of models where flow, diffusion, or autoregressive approaches outperform MCMC/Langevin. This will be the case in statistical inference problems presenting a hard phase, i.e., presenting a sharply defined region of the noise $\Delta_{\mathrm{IT}}>\Delta >\Delta_{\mathrm{alg}}$ that is computationally hard for message-passing algorithms and conjectured hard for any other efficient algorithm (45). A recent line of work (30, 49, 50, 79, 80) argues that when it comes to sampling algorithms such as Langevin or other algorithms walking in the space of configurations, such as gradient descent, the hardness actually extends to even beyond the threshold $\Delta_{\mathrm{alg}}$ up to some $\Delta_{\mathrm{MCMC}}$ that depends on the specific algorithm. Yet, diffusion models were shown to be able to sample down to $\Delta_{\mathrm{alg}}$ (37). We will also illustrate this here by studying the tilted and pinning measures of the following prototypical model:

- **Sparse rank-one matrix factorization**: The sparse spiked Wigner model and its phase diagram were discussed, e.g., in refs. 81, 82, 83, 84. This model is a variation of the “planted” Sherrington–Kirkpatrick model in spin glass physics. In such models, one is given a “spiked” version of a random symmetric matrix with a rank-one perturbation with a planted vector $x^{*}$ and aims at finding back $x^{*}$ from the matrix. This is done by sampling from the posterior probability, and therefore one considers the probability distribution:
  [20] $$P_{0}(x)\propto \underset{i}{\prod }P_{X}(x_{i})\underset{i<j}{\prod }\exp(\frac{1}{\text{Δ}\sqrt{N}}J_{ij}x_{i}x_{j}-\frac{1}{2\text{Δ}N}x_{i}^{2}x_{j}^{2}),$$
  $$\begin{array}{c} J_{ij}=\frac{x_{i}^{*}x_{j}^{*}}{\sqrt{N}}+z_{ij},\;\;\;\;\;z_{ij}=z_{ji}∼N(0,\text{Δ});\\ P_{X}(x)=(1-\rho )\delta_{x,0}+\frac{\rho }{2}\left(\delta_{x,+1}+\delta_{x,-1}\right),\;\;\;\;\;\;x_{i}^{*}∼P_{X}\;\forall i. \end{array}$$

## Phase Diagrams of the Tilted and Pinning Measures

We first discuss sampling in the spherical p-spin model, Eq. **18**. We focus in particular on sampling in its paramagnetic phase, i.e., $T>T_{d}$, where MCMC and Langevin algorithms are predicted to work efficiently. In order to analyze flow-based, diffusion-based, and autoregressive sampling, we need to consider the corresponding denoising problems. For flow-based and diffusion-based sampling, this leads to the tilted measure Eq. **11**, with $P_{0}$ now given by Eq. **18**. Taken together, this defines a variant of the p-spin model with a particular random field. While the tilted measure may look complicated at first sight, because of the random field in the direction of a particular “equilibrium” direction $x_{0}$, it turns out that measures of this type were already studied in the literature. We refer the reader to *SI Appendix*, section 1 for details and only briefly sketch the reasoning here. The trick (also used in, e.g., refs. 36 and 85) is to notice that $\forall T>T_{K}$ the original p-spin model is contiguous to its planted version, where a vector $x_{0}$ has been hidden beforehand as an equilibrium configuration [the spike tensor model (86)]. The model is thus equivalent for all practical purposes to its planted version, with the tilted field now acting in the direction of the planted configuration.

Computing the phase diagram of the model associated with the tilted measure thus requires computing the free entropy of the planted model with an additional side Gaussian information. This is the same computation as needed in various mathematical works based on Guerra’s interpolation technique where the planted model is observed together with an additional Gaussian channel, e.g., in ref. 32. This, and the single-state property of Bayes-optimal denoising discussed in the former section (that ensures replica symmetry), allows us to obtain the phase diagram of the tilted measure. For the present spherical p-spin model, we show in *SI Appendix*, section 3 that the equilibrium properties for $T>T_{K}$ are given by

[21] $$\begin{array}{cc} \chi^{*} & =\underset{\chi }{\mathrm{argmax}}\;\Phi_{\mathrm{RS}}\left(\chi \right),\\ \Phi_{\mathrm{RS}}\left(\chi \right) & =\frac{\tilde{\chi }}{2}+\frac{1}{2}\log\left(\frac{2\pi }{\tilde{\chi }+1}\right)-\frac{1}{2T^{2}}\chi^{3},\\ \;\tilde{\chi } & =\frac{3}{2T^{2}}\chi^{2}+\gamma^{2}. \end{array}$$

**Fig. 2.**
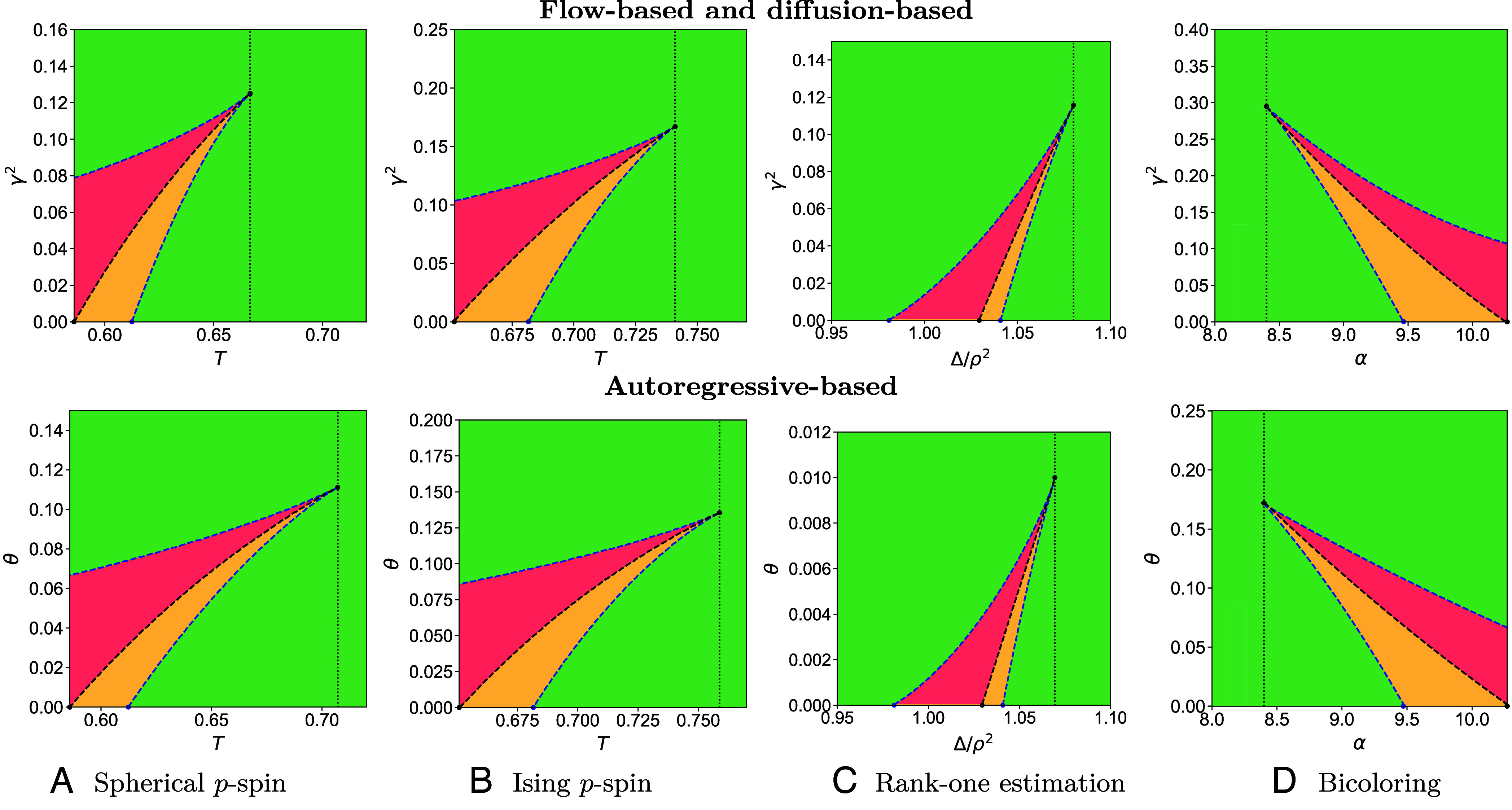
Phase diagrams for the tilted measure $P_{\gamma }$ (*Top*), and the pinning measure $P_{\theta }$ (*Bottom*): (*A*) The spherical p-spin model with P=3, (*B*) the Ising p-spin model with P=3, (*C*) the sparse rank-one matrix estimation with ρ=0.08, (*D*) the k-bicoloring problem on hypergraphs (NAE-SAT) with k=5. The *x*-axis is the temperature T=1/β in (*A*) and (*B*), the inverse-SNR $\Delta /\rho^{2}$ in (*C*) and the clauses-to-variables ratio α in (*D*), while the *y*-axis shows the SNR ratio $\gamma^{2}=\alpha^{2}/\beta^{2}$ (*Top*) and the decimated ratio θ (*Bottom*). In the green phase, there is a single maxima to the free entropy. The red and orange regions display a phase coexistence with two maxima. In the red region, efficient denoising is predicted to be algorithmically hard. In (*A*) the spherical p-spin at γ=θ=0, the dynamical threshold $T_{d}=\sqrt{3/8}$, the Kauzmann transition $T_{K}\approx 0.58$, while the tricritical point is at $T_{\mathrm{tri}}=2/3$ for diffusion and $T_{\mathrm{tri}}=\sqrt{1/2}$ for autoregressive. In (*B*) the Ising p-spin the values are $T_{d}\approx 0.682$, $T_{K}\approx 0.652$, $T_{\mathrm{tri}}\approx 0.741$ for diffusion, and $T_{\mathrm{tri}}\approx 0.759$ for autoregressive. In (*C*), for the sparse rank-one matrix estimation at ρ=0.08, we have $\Delta_{d}/\rho^{2}\approx 1.041$, $\Delta_{K}/\rho^{2}\approx 1.029$, and $\Delta_{\mathrm{alg}}/\rho^{2}\approx 0.981$. The tricritical points are at $\Delta_{\mathrm{tri}}/\rho^{2}\approx 1.08$ for diffusion, and $\Delta_{\mathrm{tri}}/\rho^{2}\approx 1.069$ for autoregressive. In (*D*), for the bicoloring problem the values are $\alpha_{d}\approx 9.465$, $\alpha_{K}\approx 10.3$. The tricritical points are $\alpha_{\mathrm{tri}}\approx 8.4$ for both diffusion and autoregressive. The curves for bicoloring were obtained by a polynomial fit, while in all the other cases, we represent directly the data points. Moreover, in *SI Appendix*, section 2, we show some examples of sampling simulations for the bicoloring and the spherical p-spin models.

Solving the above maximization problem is easily done. One observes that depending on the range of parameters T and γ, up to two local maxima of $\Phi_{\mathrm{RS}}$ can be found for this model. In Fig. 2*A*, *Top*, we depict in green the regions of T,γ where $\Phi_{\mathrm{RS}}$ has a unique maximizer. The orange region is where two maximizers coexist with the global one having a smaller value of the order parameter χ, and in the red region, the global maximizer has a larger value of χ. Such a phenomenology is familiar in first-order phase transitions, where the red and orange phases in Fig. 2 correspond to the phase coexistence region.

Now, a crucial point for the follow-up discussion of the flow- and diffusion-based sampling is that it is widely believed to be algorithmically hard to obtain the Bayes-optimal denoiser in the red region for all efficient algorithms (even if $P_{0}$ is known) (23, 45, 48, 52). This is nothing else than the well-known metastability problem in thermodynamics, as one is “trapped” in the wrong maxima of Eq. **21**.

The evidence for denoising being algorithmically hard in the red region goes beyond mere physical analogies and is an intense subject of studies in computer science, with the study of a variety of techniques such as low degree polynomial or message-passing algorithms (see, e.g., ref. 48). Note that while denoising is hard in the red region, direct sampling with MCMC is hard already in the orange one.

To explain this further, let us discuss a concrete implementation of a denoiser. Computing the marginals for the tilted measure is a classical topic in spin glass and estimation theory, and the best-known algorithm to do it efficiently is the so-called mean-field Thouless–Anderson–Palmer equations (51), or—to use their modern counterpart—the iterative approximate message passing (AMP) (31, 87). The AMP algorithm is an iterative update procedure on the estimates of the posterior means ${\hat{x}}_{i}$ and the covariances $\sigma_{i}$, and for the spherical 3-spin model it reads

$$\begin{array}{c} \left\{\begin{array}{c} B_{i}^{t}=\frac{\sqrt{3}\beta }{N}\sum_{j<k}J_{\mathrm{ijk}}{\hat{x}}_{j}^{t}{\hat{x}}_{k}^{t}-\frac{3}{N}\beta^{2}{\hat{x}}_{i}^{t-1}\sigma^{t}{\hat{x}}^{t}\cdot {\hat{x}}^{t-1}\\ {\hat{x}}_{i}^{t+1}=\frac{B_{i}^{t}+\frac{\alpha (t)}{\beta {\left(t\right)}^{2}}{\left[Y_{t}\right]}_{i}}{3\beta^{2}{\left\|\hat{x^{t}}\right\|}_{2}^{2}/\left(2N\right)+\gamma^{2}+1}\;,\sigma^{t+1}=\frac{1}{3\beta^{2}{\left\|\hat{x^{t}}\right\|}_{2}^{2}/\left(2N\right)+\gamma^{2}+1}. \end{array}\right. \end{array}$$

The virtue of AMP is that its performance can be tracked rigorously over iteration time, and in fact, one can show that the overlap $\chi^{t}$ defined by the AMP estimates obeys the following state evolution:

[22] $$\begin{array}{c} \chi^{t+1}=\frac{\tilde{\chi^{t}}}{1+\tilde{\chi^{t}}}\;,\;{\tilde{\chi }}^{t}\equiv \frac{3}{2T^{2}}{\left(\chi^{t}\right)}^{2}+\gamma^{2}, \end{array}$$

which is nothing but the fixed point equation of Eq. **21**. We see now how the presence of multiple fixed points can trap the algorithm in the wrong maximizer. Turning an AMP-denoiser into a sampler was precisely the idea introduced in refs. 37 and 42 under the framework of stochastic localization (33–35). While we derived the equation for a flow-based approach, the conclusion of refs. 37 and 42 remains. In particular, leveraging the rigorous analysis of the asymptotic error obtained by AMP (88, 89), they prove that AMP can approximate optimal denoising throughout the interpolation path in the regime where the global maximum for $\Phi_{\mathrm{RS}}$ is the first one reached by the state evolution (37). Furthermore, using the local convexity of the TAP free energy (36, 90), they show that the AMP iterates satisfy Lipschitz-continuity w.r.t the observation $Y_{t}$. This is crucial for the SDE-based sampling in refs. 36 and 37 as well as the continuous flow-based sampler in our case, since both require control of the discretization errors. We discuss this further in *SI Appendix*, section 5. However, AMP and, conjecturally (45), any other polynomial-time denoiser (and in particular any neural-network denoiser learned from data) fail to return the correct marginal whenever the global maxima of $\Phi_{\mathrm{RS}}$ is not the first one encountered by the state evolution, i.e., in the red region in Fig. 2.

### Failure of Generative Models While MCMC Succeeds.

Recall now how the flow- or diffusion-based methods use the denoiser (the marginal of the tilted measure) to produce samples from $P_{0}$. They start with a Gaussian noise at γ=0 and increase γ gradually while transforming the Gaussian noise toward the direction of the marginal of the tilted measure. To do this for a specific temperature T, one needs to be able to denoise optimally on a vertical line at all values of γ∈[0,∞[. However, for temperatures below the tricritical points $T_{\mathrm{tri}}=2/3$ (this value is for diffusion, in the spherical p-spin model) we see that we encounter the first-order phase transition, and the metastable region (red in Fig. 2) where optimal denoising is computationally hard and hence the uniform sampling using this strategy is as well. Another, less critical, problem is the fact that the measure will change drastically at the phase transition (as the value $\chi^{*}$ jumps discontinuously) which means one has to be very careful with the discretization of the diffusion process. Based on this reasoning, we interpret the phase diagram of the tilted measure as a representation of the presence of a fundamental barrier for sampling by flow- and diffusion-based methods: the sampling scheme corresponds to starting from γ=0 and going upward. If this path intersects the hard phase (red) at any time, it means that the Bayesian denoising cannot be performed optimally in an efficient way, thus preventing the sampling scheme from working efficiently in consequence. In particular, for the spherical p-spin model, we computed the curves analytically; see *SI Appendix*, section 3, and found that this hurdle is present at temperatures up to the tricritical point $T_{\mathrm{tri}}=2/3$, strictly larger than the dynamical temperature $T_{d}=\sqrt{3/8}$ (threshold between the orange and red regions at γ=0) down to which MCMC and Langevin algorithms are predicted to sample efficiently in the literature.

Indeed, one can write exact equations describing the Langevin dynamics (25) [and prove them (27)]. The analysis shows that Langevin is efficient at sampling for all $T>T_{d}$ (91). On the other hand, for $T<T_{d}$, we are in the so-called “dynamical” spin glass phase and Langevin fails to sample in linear time, this is the aging regime (25, 26). In fact, all polynomial algorithms are conjectured to fail to sample efficiently, as can be proven for any “stable” algorithm (72).

The situation for sampling with autoregressive network, analyzed via the phase diagram of the pinning measure, is very analogous; see the lower row of Fig. 2. The pinning measure again defines a variation of the original p-spin model, and as shown in *SI Appendix*, we can compute properties at equilibrium by solving $\chi^{*}={\mathrm{argmax}}_{\chi }\;\Phi_{\mathrm{RS}}\left(\chi \right)$ where the RS free entropy reads

[23] $$\begin{array}{cc} \Phi_{\mathrm{RS}}\left(\chi \right) & =\frac{\tilde{\chi }}{2}+\frac{1-\theta }{2}\log\left(\frac{2\pi }{\tilde{\chi }+1}\right)-\theta \log\left(\sqrt{2\pi }e\right)-\frac{\chi^{3}}{2T^{2}},\\ \tilde{\chi } & =\frac{3\chi^{2}}{2T^{2}}. \end{array}$$

The same reasoning can be applied to this phase diagram (and in fact the so-called decimated versions of message-passing algorithms were proposed as early as in refs. 54 and 55). Concerning the efficiency of sampling, the same phenomenology appears again when interpreting this phase diagram. In fact, for the spherical p-spin model, it turns out to be even worse because the temperature where the difficulty arises for the autoregressive method is $T_{\mathrm{tri}}=\sqrt{1/2}$, which is larger than the one for flow-based and diffusion models $T_{\mathrm{tri}}=2/3$. So in this case, autoregressive-based sampling algorithms perform worse than flow- or diffusion-based.

### Other Models.

Fig. 2 then evaluates the phase diagrams of the tilted and pinning measures for three other models—the Ising p-spin (*B*), the rank-one matrix estimation (*C*), and the bicoloring problem on sparse hypergraphs (*D*). For the bicoloring problem, defined on random sparse hypergraphs, we can use the belief propagation equations as Bayesian denoiser (73–75). The resulting equations are quite long, and we defer their presentation in *SI Appendix*, along with their derivation using the cavity method.

We observe the same phenomenology with tricritical points causing hurdles for flow, diffusion, and autoregressive methods reaching out to the phase where traditional approaches based on MCMC or Langevin work efficiently. In fact, we expect this picture to always appear for any model with the RFOT phenomenology where the dynamical temperature is distinct from the ideal glass or Kauzmann temperature. Such a phenomenology was described in many problems far beyond those we picked to study, and we can hence anticipate follow-up studies identifying analogous phase diagrams in many other problems of interest.

Finally, we notice that depending on the model, the position of the tricritical point for flow- and diffusion-based methods is better (e.g., for the spherical 3-spin, Ising 3-spin) or worse (e.g., for the sparse rank-one matrix estimation) than for the autoregressive methods. In any case, the position of the tricritical point does depend on the noise channel to which the generative model maps. This leaves open the question of which channel one should use, for each model, to minimize the range of values for which generative model-based sampling is suboptimal compared to MCMC techniques that fail at the dynamical threshold $T_{d}$. It is not inconceivable that it is possible to reduce $T_{\mathrm{tri}}$ very close (or maybe even up to) $T_{d}$ by optimizing over different distributions in linear interpolant (41), or using nonlinear maps (37). This is left for future studies.

### Outperforming MCMC in Inference Models with a Hard Phase.

We now discuss a situation more advantageous for modern techniques. Column (c) of Fig. 2 depicts the phase diagram of the sparse rank-one matrix estimation problem that presents an additional interesting feature: here, there is a planted “hidden” signal that we seek to recover. At large values of the noise the signal is hidden and the RFOT-type phenomenology reappears so that the high noise behavior is identical to the high-temperature models of the other models.

At low noise values, however, there is another phase transition at γ=θ=0 denoted as $\Delta_{\mathrm{alg}}$ below which the AMP algorithm solves the estimation problems optimally and above which it does not up to the value $\Delta_{\mathrm{IT}}$ (the hard region, in red, is delimited by these two values). Going vertically up in the phase diagram in γ or θ for $\Delta <\Delta_{\mathrm{alg}}$ does not cause any encounter of the hard (red) phase, and thus sampling based on flow or diffusion or autoregressive networks works. This has been proven rigorously recently in ref. 92 [with some technical regularity assumptions on the denoiser (90)].

Yet existing literature collects evidence that in inference problems that present such a hard phase, local dynamics algorithms such as MCMC and Langevin are not able to sample efficiently until some yet lower values of noise $\Delta_{\mathrm{MCMC}}$. In particular, this suggestion was put forward indirectly in ref. 30 by arguing that the metastable phase in the hard region is glassy, and this glassy nature extends well beyond the region that is hard for message-passing algorithms such as AMP. This was then shown explicitly in follow-up works starting with an analysis of the dynamics in a mixed spiked matrix-tensor model in ref. 49, in the phase retrieval problem (79), the planted coloring (50), and on a rigorous basis in the planted clique problem (80).

In light of these works, it is interesting to note that sampling based on flow, diffusion, or autoregressive networks also avoids hurdles stemming from the glassiness of the hard phase and rather effortlessly so by working in the space of marginals rather than configurations directly. The phase diagram presented in Fig. 2 indicates that both diffusion and autoregressive networks sample the $P_{0}$ efficiently for any $\Delta <\Delta_{\mathrm{alg}}$. This poses an intriguing question for future work of whether overparameterization of neural networks that are learning the denoisers from data would still be so beneficial in these methods.

Finally, we comment on the relevance of these findings beyond the specific model discussed here, and in particular for the study of physical objects in finite dimensions with short-range interaction. Do we expect a problem embedded in a finite dimension to suffer the same fate as the one discussed here? While similar phase diagrams as Fig. 2 have been observed in finite dimension in, e.g., refs. 70 and 93, the phenomenology of first-order transition is different. From nucleation arguments, the exponentially hard denoising phase is not expected to exist in finite dimension (94). Indeed, it can be proven rigorously that for any graph that can be embedded in a finite-dimensional lattice, an efficient algorithm exists for the problems considered here (95). In this case, the analysis of whether a good denoiser can be learned with a neural network will require a finer study, depending on the discretization of the ODE close to the transition, and on the number of points in the dataset (perhaps in the vein of ref. 57). These are interesting potential directions of research.

## Conclusion

Our investigation into the efficiency of sampling with modern generative models, in comparison with traditional methods, reveals distinct strengths and weaknesses for each. By examining a specific class of probability distributions from statistical physics, we identified parameters where either method excels or falls short. Significantly, our approach highlighted challenges stemming from a first-order discontinuous transition for generative models-based sampling techniques even in regions of parameters where traditional samplers work efficiently. While generative models have shown promise across various applications, it is crucial to understand their potential pitfalls and advantages in specific contexts, and our paper makes a key step in this direction.

## Supplementary Material

Appendix 01 (PDF)

## Data Availability

There are no data underlying this work.
