# Supplementary material for: Sampling with flows, diffusion, and autoregressive neural networks from a spin-glass perspective
Source: Proc Natl Acad Sci U S A. 2024 Jun 24;121(27):e2311810121. doi: 10.1073/pnas.2311810121 (PMC11228464; doi:10.1073/pnas.2311810121)
Supplement: Supplementary file 1 — Appendix 01 (PDF) [file pnas.2311810121.sapp.pdf]

Supporting Information for

Sampling with flows, diffusion and autoregressive neural networks from a spin-glass perspective

Davide Ghio, Yatin Dandi, Florent Krzakala and Lenka Zdeborová

Lenka Zdeborová.
E-mail: lenka.zdeborova@epfl.ch

This PDF file includes:

Supporting text
Figs. S1 to S11
SI References

Supporting Information Text

This Supporting Information Text is organized as follows. In Section 1 we describe the mathematical properties we have exploited to simplify the study of the tilted and pinning measures presented in the main text; namely the contiguity to a planted version of the statistical physics model under consideration. In Section 2 we then show some numerical simulations of the sampling algorithm to support the claim that indeed it stops working under certain conditions. Section 3 is devoted to the derivation of the asymptotic solutions for all the models mentioned in the paper, along with the corresponding phase diagrams. In Section 4 we remind some well-known properties of probability and statistics that we have used in the main text and, finally, in Section 5 we present a theoretical analysis of the sampling scheme presented in Algorithm 1.

1. Computing free entropies: contiguity to the planted model

Let us start by reminding the expression of the tilted measure

$$P_\gamma(\mathbf{x}|\mathbf{x}_0, \mathbf{z}) \propto e^{\gamma(t)^2\langle \mathbf{x}, \mathbf{x}_0\rangle + \gamma(t)\langle z, \mathbf{x}\rangle - \frac{\gamma(t)^2}{2}\|\mathbf{x}\|^2} P_0(\mathbf{x}), \qquad [1]$$

with P_0 a given probability measure. The main technical difficulty in the study of this measure is the presence in the exponential of $\mathbf{x}_0$, which is an equilibrium configuration and hence in general it is correlated with $\mathbf{x} \sim P_0$. In the following, we explain how this difficulty can be avoided, by considering a "planted" version of our models, meaning that a hidden assignment of the variables is planted in the model as an equilibrium configuration.

While this is already the case for actual inference statistical models (such as sparse rank-one matrix factorization), it turns out that the p-spin (1) and the bicoloring (2) models are contiguous to their planted version as long as $T > T_K$ (for p-spin) or $\alpha < \alpha_K$ (for bicoloring). For p-spin models, such contiguity has also been rigorously established in certain setups (3–5).

Therefore, by studying planted models, the difficulty previously discussed is greatly simplified, as the joint distribution of the disorder and an equilibrium configuration $\mathbf{x}_0$ can be substituted with the joint distribution of the disorder and the planted configuration.

This property, along with the previously discussed contiguity of the models we study to their planted version, allows us to replace the equilibrium configuration $\mathbf{x}_0$ in the tilted measure in Eq. [1] with the planted vector, thus simplifying considerably the analysis of the resulting problem.

For the case of the Sherrington-Kirkpatrick (SK) model, in (6) such an equivalence was rigorously proven and we refer the interested reader to Section 4 there for the mathematical details. Informally, Proposition 4.2 in (6) shows the equivalence between the following two methods for generating the tilted measure:

1. First sample $\mathbf{x_0}$ uniformly and then generate the interaction matrix as $J = \frac{\beta}{n}\mathbf{x_0}\mathbf{x_0}^\top + W$ where $W \sim \text{GOE}$.
2. First sample $J \sim \text{GOE}$ and then generate the equilibrium configuration $\mathbf{x_0} \sim P_{\text{SK}}(J)$,

where $P_{\text{SK}}(J)$ denotes the Boltzmann Sherrington-Kirkpatrick measure with interaction matrix J.

Similarly, based on the contiguity between planted and random models, we assume that the above equivalence holds for $T > T_K$ (for p-spin) and for $\alpha < \alpha_K$ (for bicoloring). We note that for inference problems, i.e. when J is planted for both points (1) and (2) above, the equivalence holds directly based on the definition of the posterior measure.

From a Bayesian point of view, the tilted measure can also be seen as the measure associated to an inference problem. Consider for concreteness the planted p-spin, also called the spiked tensor model (4, 7), in which one extracts a random vector $\mathbf{x_0} \in \mathbb{R}^N$ from a Gaussian or a Rademacher distribution, and then one aims at recovering $\mathbf{x_0}$ from a Gaussian measurement $\mathbf{y}(t) = \alpha(t)\mathbf{x_0} + \beta(t)\mathbf{z}$ plus a noisy tensor measurement $J_{ijk} = \frac{1}{n\Delta}[\mathbf{x_0}]_i[\mathbf{x_0}]_j[\mathbf{x_0}]_k + \xi_{ijk}\ \forall i < j < k$. The Bayesian posterior describing this model is nothing but the tilted measure (Eq. [1]) applied to the spiked tensor model.

Interestingly, such considerations are not new. Nishimori (8) and later Iba (9) already used a similar trick, and (10–12) used it to discuss the dynamics starting from equilibrium conditions, while (13) used it in the context of error correction, all for the p-spin model. Recently, the same technique was used to prove the clustering property in the p-spin model (14).

Adding a Gaussian measurement to an inference problem is also a classical trick used when proving free energies in the mathematical physics literature, especially in the context of Guerra interpolation (15, 16) for Bayes optimal models, see e.g. (17–20), and thus such free energies have been solved rigorously as well.

In the case of the decimated problem, the additional Gaussian channel is replaced by an erasure channel and we called the resulting probability the pinning measure, which we can use to analyse the autoregressive-based sampling procedure:

$$P_\theta(\mathbf{x}|\mathbf{x}_0, S_\theta) \propto P_0(\mathbf{x}) \prod_{i \in S_\theta} \delta([x_i - [\mathbf{x}_0]_i) \qquad [2]$$

where θ is the fraction of pinned variables and S_θ is the set of revealed variables, of size θN.

Again, this is precisely one of the alternatives used in the mathematical physics literature to prove free energies. Indeed, the pinning lemma (21) (see also (22)) is often used instead of the Gaussian channel.

These considerations are really helpful, as we can now study the performance of generative models as a simple variant of problems already solved in the literature, often rigorously (in the case of the p-spin we refer to (23) and (24), and for the spiked Wigner model to (25–27)).

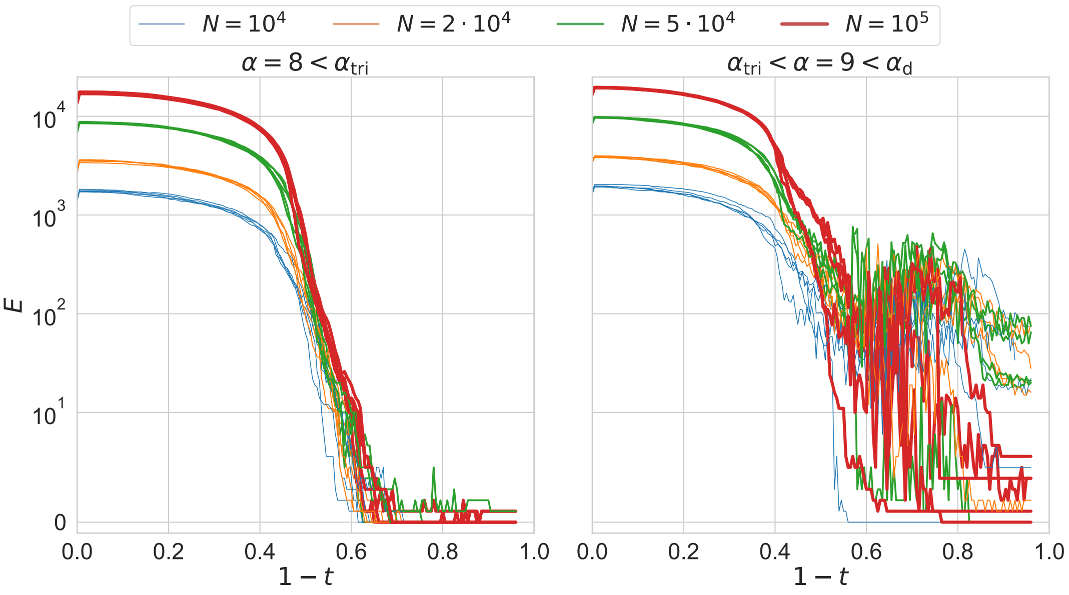

Fig. S1. **k-NAESAT with $k=5$: Energy vs sampling time for flow-based sampling.** We compute the energy of the configuration selected by Algorithm 1 versus the sampling time, for simulations with different sizes $N \in [10^4, 10^5]$. We performed 5 simulations for each value of N. The y-axis in both panel is in *symlog*, meaning that data is plotted in linear scale for $E \in [0, 10^1]$ and in logarithmic scale for $E > 10^1$. Left: $\alpha = 8 < \alpha_{\mathrm{tri}}$, we show that the algorithm is able to converge to configurations of zero energy for all sizes N and almost all instances of the simulation. Right: $\alpha_{\mathrm{tri}} < \alpha = 9 < \alpha_d$, conversely, we show that the majority of instances the configuration selected by BP has non-zero energy and thus we are clearly not sampling the model, since we are considering the zero temperature case, and thus the correct energy should be zero.

Moreover, since these are variations of known inference problems, we can leverage on the existing work on approximate message passing (28) for the p-spin model (7, 23) and the spike model (25), see in particular (4) for a detailed presentation.

Finally, for problems defined on sparse graphs, e.g. bicoloring considered in the paper, a rigorous control is harder, and thus we shall simply stay at the level of rigour of the cavity method (29) and use the results of (30). Note however that the method is trustworthy (2).

2. Sampling simulations for Algorithm 1

In this section, we present some finite-size simulations of Algorithm 1, mainly focusing on comparing its behavior at high temperatures, where we expect it (from the results in the main text) to work, to the case in which it encounters a first order transition in the denoising path, where instead we expect it not to be able to sample.

We start by considering the k-NAEAT model, with $k=5$, where we are able to reach a considerably big size N. In general, when the algorithm is working, we expect the single node marginals of Belief Propagation to concentrate more and more on a single value during sampling, until at time $t \approx 0$ we can extract a configuration accordingly, which will be a correct sample of the model.

One necessary condition we can look at to check it is indeed a sample is the energy of the final configuration, which in the case of k-NAESAT is simply the number of unsatisfied clauses. Since we are looking at the zero-temperature version of the model, a sample needs at least to be a solution of the constrained satisfaction problem, i.e. the configurations energy should be zero.

In Fig. S1 we compute this energy E through the different sampling steps, considering as configuration the most probable one according to the BP marginals. On the left panel, we show what happens for $\alpha = 8$, which is low enough for the alogithm to work well. Indeed, we see that in this case for almost all instances the energy decreases during denoising until reaching $E = 0$, usually even much before reaching $t = 0$. The picture is strikingly different in the right panel, where instead we look at the case $\alpha = 9$, which is still lower than the dynamical-1RSB transition, but now it is higher than α_{tri}, where a first order transition starts to appear in the denoising path. We see how this has consequences on the simulations: while at the beginning the energy curve seems to follow a very similar path compared to the left panel, at some point (for the vast majority of the simulations) the curves stop decreasing. At this point, the energy starts oscillating around a value $E \neq 0$ and remains positive until the end and thus we can say that the algorithm is not able to sample correctly the model, corroborating the theoretical results presented in the main text.

In Fig. S2 we report some numerical simulations where we compare the asymptotic curves derived through state evolution with some empirical simulations implementing the flow-based sampling for the spherical p-spin model.

Specifically, we compare what happens at a high value of temperature $T > T_{\mathrm{tri}}$, where we expect the sampling scheme to work, to a value of T in the interval $[T_d, T_{\mathrm{tri}}]$, where, as previously explained, we predict the algorithm to fail.

In the first case, shown in the left part of the plot, we see that finite-size simulation follows very well the theoretical prediction, already at a considerably low number of variables. Conversely, in the second case, reported in the right part, the

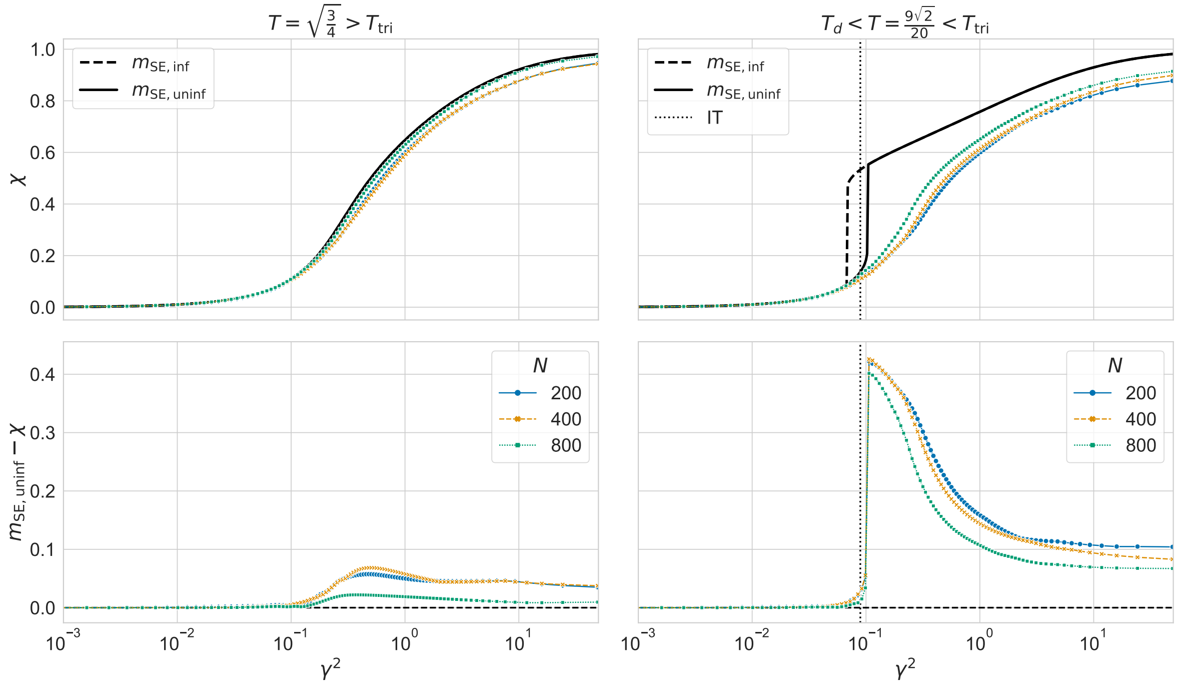

Fig. S2. Spherical p-spin with $p=3$: $\chi(\gamma)$ for flow-based sampling. We compare the results for the order parameter χ computed from the State Evolution equations (black lines) to finite-size implementations of the sampling algorithm in two regimes. Left: $T=\sqrt{\frac{3}{4}}>\Delta_{\rm tri}$, for all values of γ^2, the SE equations have a unique fixed point, and thus the initialization plays no role. The resulting curve (black continuous line) is compared to algorithmic implementations of the flow-based sampling algorithm, for sizes $N=200,400,800$. These curves, shown in different colours above, match the asymptotic prediction. Right: $T_d<T=\frac{9\sqrt{2}}{20}<T_{\rm tri}$, there is a range of values of γ^2, for which the SE equations have two distinct fixed points. More precisely, for all values between the IT point (reported as a dotted line) and the informed spinodal point the model presents an algorithmically hard phase. The uninformed/informed state evolution curves (black continuous/dashed lines respectively) are compared to algorithmic implementations of the flow-based sampling algorithm, for sizes $N=200,400,800$. Importantly, we show that in this regime there is an evident mismatch with the asymptotic prediction.

situation is different, and the curves show a gap.

Moreover, we check when the Nishimori conditions are satisfied for different values of temperature T in the spherical p-spin. We do this by analysing the following observable:

$$\mathrm{OV}(\gamma)\equiv\frac{1}{N}\mathbb{E}\left[\mathbf{Y_t}\langle\mathbf{x}\rangle\right] \qquad [3]$$

which is the overlap between the observation vector $\mathbf{Y_t}$ and the average magnetization $\langle\mathbf{x}\rangle$.

Indeed, by definition of the observation process we can write

$$\mathrm{OV}(\gamma)=\frac{\alpha(t)}{N}\mathbb{E}\left[\mathbf{x_0}\langle\mathbf{x}\rangle\right]+\frac{\beta(t)}{N}\mathbb{E}\left[\mathbf{z}\langle\mathbf{x}\rangle\right] \qquad [4]$$

and furthermore by using Stein's lemma (31) we can write it as

$$\begin{aligned}\mathrm{OV}(\gamma)&=\frac{\alpha(t)}{N}\mathbb{E}\left[\mathbf{x_0}\langle\mathbf{x}\rangle\right]+\frac{\beta(t)}{N}\mathbb{E}\left[\partial_{\mathbf{z}}\langle\mathbf{x}\rangle\right]\\&=\frac{\alpha(t)}{N}\mathbb{E}\left[\mathbf{x_0}\langle\mathbf{x}\rangle\right]+\frac{\beta(t)}{N}\mathbb{E}\left[\frac{\alpha(t)}{\beta(t)}\langle\|\mathbf{x}\|_2^2\rangle-\frac{\alpha(t)}{\beta(t)}\langle\mathbf{x}\rangle^2\right]\\&=\alpha(t)\frac{1}{N}\mathbb{E}\left[\mathbf{x_0}\langle\mathbf{x}\rangle+\langle\|\mathbf{x}\|_2^2\rangle-\langle\mathbf{x}\rangle^2\right]\approx\alpha(t)+\alpha(t)\frac{1}{N}\mathbb{E}\left[\mathbf{x_0}\langle\mathbf{x}\rangle-\langle\mathbf{x}\rangle^2\right]\,.\end{aligned} \qquad [5]$$

where in the last step we used the fact that $\lim_{N\to\infty}\|\mathbf{x}\|_2^2/N=1$. Now, *if* the Nishimori conditions are satisfied, we have that $\mathbb{E}\left[\mathbf{x_0}\langle\mathbf{x}\rangle\right]=\mathbb{E}\left[\langle\mathbf{x}\rangle^2\right]$. Putting this equivalence back into Eq. [5], we see that in this case OV coincides with the function $\alpha(t)$ that defines the interpolant process.

In Fig. S3, we use this equivalence and compare with $\alpha(t)=1-t$, the behaviour of OV for $T=\frac{9\sqrt{2}}{20}>T_{\rm tri}$ and for $T_d<T=\sqrt{\frac{3}{4}}<T_{\rm tri}$, showing that in the first case we are Bayes optimal for all the values of γ, and thus at each sampling step. Instead, in the second case, even if at the beginning the two curves coincide, around the IT threshold they develop a gap, and after this point they take two different paths. This behaviour is consistent with what we observed from the study of the order parameter χ, for which also a gap with the state evolution equations was developed around the IT threshold.

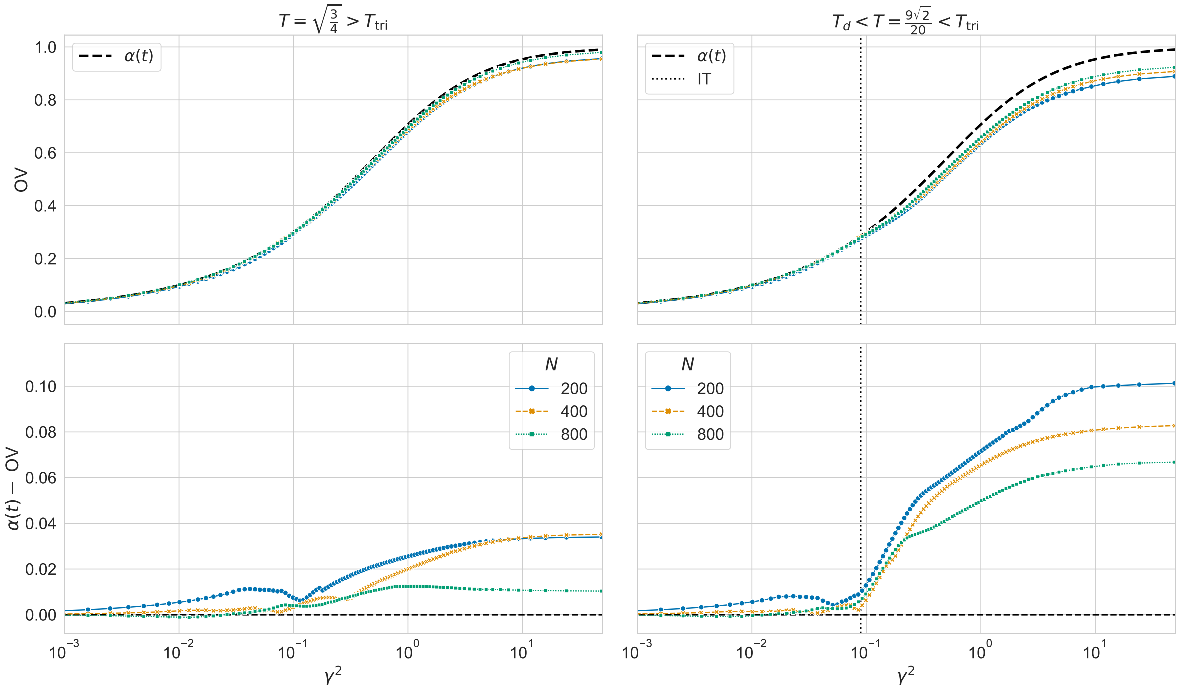

Fig. S3. Spherical p-spin with $p = 3$: Checking the Nishimori conditions for flow-based sampling. We compare the results for the overlap OV, defined in Eq. [3] computed from finite-size ($N = 200, 400, 800$) implementations of the sampling algorithm to the behaviour with γ of the function $\alpha(t) = 1 - t$ in two regimes. Left: $T = \sqrt{\frac{3}{4}} > \Delta_{\mathrm{tri}}$. The simulations, shown in different colours above, are very close to the asymptotic prediction, even if the sizes are relatively limited. Right: $T_d < T = \frac{9\sqrt{2}}{20} < T_{\mathrm{tri}}$. Here, after the IT threshold there is an evident mismatch between the two curves, due to the departure of the algorithm from the Bayes-optimality regime.

3. Asymptotic solutions and Phase Diagrams

In this section, we present how each of the phase diagrams we presented in the main text can be produced. Specifically, we first remind our definition of tilted and pinning measures and of the order parameters we are going to use for the analysis. After defining the probability distribution associated to each problem, we give the expression of the RS free entropy, from which one can compute the values of the parameters of the problem at which the *spinodal points* are located, i.e. the points at which the potential develops a second maxima by continuous deformation, and also the point corresponding to the *IT transition*, i.e. where the two maxima exchange the role of global and local maxima.

We then report the expression of the denoisers (AMP/BP) used in the sampling schemes illustrated in the main text, with their associated self-consistent asymptotic equations (state evolution for AMP and Cavity equations for BP). Looking at the fixed points of these equations, starting from an uninformed and informed initialization, we can plot the difference between the values of the order parameter reached at the fixed point in these two cases, which allows detecting the phases in which multiple fixed points are present.

In Eq. [1] and Eq. [2] we have recalled the expression for the tilted measure and the pinning measure, characterizing the flow-based sampling scheme and the autoregressive scheme respectively. As we already mentioned, γ is the rescaled sampling time, while θ is the fraction of revealed variables, such that studying the properties of the tilted measure varying γ/θ allows us to characterize the properties of the Bayesian denoising problem at all times during sampling.

Finally, let us remind that we shall study the evolution in time (or equivalently in $\gamma \in [0, \infty[$ (AWGN) and $\theta \in [0, 1]$ (BEC)) of the following order parameters:

$$\mu(\gamma) \equiv \frac{1}{N}\mathbb{E}[\hat{\mathbf{x}}(\gamma) \cdot \mathbf{x}_0]\,, \qquad [6]$$

$$\chi(\gamma) \equiv \frac{1}{N}\mathbb{E}[\|\hat{\mathbf{x}}(\gamma)\|^2]\,, \qquad [7]$$

and analogously we can define $\mu(\theta)$ and $\chi(\theta)$. Concretely, we will consider only cases in which the Nishimori identities hold, such that these two quantities always coincide, and thus we will restrict our analysis to χ.

Let us now go through each one of the models mentioned in the main text.

A. Sparse rank-one matrix factorization. We consider the Bayes-Optimal rank-one matrix estimation (or rank-one matrix factorization) problem:

Given a hidden vector $\mathbf{x}^*$, sampled from the so-called *Rademacher-Bernoulli* prior distribution

$$P_X(x) = (1-\rho)\delta_{x,0} + \frac{\rho}{2}\left(\delta_{x,+1} + \delta_{x,-1}\right), \quad x_i^* \sim P_X\ \forall\, i\,.$$

one has access to noisy observations, that is a matrix J_{ij} is composed by a rank-one spike plus i.i.d. Gaussian noise:

$$J_{ij} = \frac{x_i^* x_j^*}{\sqrt{N}} + \widetilde{z}_{ij}\,, \quad \widetilde{z}_{ij} = \widetilde{z}_{ji} \sim \mathcal{N}(0,\Delta)\,;$$

and the goal is to infer $\mathbf{x}^*$ in the best way possible. There are many important problems in statistics and machine learning that can be expressed in this way (32), and this model has been the subject of many works both from the statistics (25, 27, 33–35) and the statistical physics communities (23, 36). The presentation of this problem closely follows the study presented in (23).

From the Bayesian point of view, the problem amounts to sampling from the posterior. One way to introduce the model is through the following probability distribution:

$$P_0(\mathbf{x}) \propto \prod_i P_X(x_i) \prod_{i<j} \exp\left(\frac{1}{\Delta\sqrt{N}} J_{ij} x_i x_j - \frac{1}{2\Delta N} x_i^2 x_j^2\right). \tag{8}$$

With this distribution, $\mathbf{x}$ will be a random vector with, on average, a fraction ρ of components that are Ising spin variables (i.e. each x_i takes values ± 1) and the rest of the entries that are put to zero, in such a way that the parameter ρ controls the sparsity of the vector we want to retrieve.

As discussed in Appendix 1, the **tilted measure** with an equilibrium vector $\mathbf{x}_0$ is equivalent to the one where the vector $\mathbf{x}_0$ is planted. Therefore, in what follows, we shall assume that $\mathbf{x}_0$ corresponds to the planted configuration. We thus obtain the following tilted measure for diffusion and flow-based models:

$$P_\gamma(\mathbf{x}) \;=\; \frac{1}{Z_\gamma}\left(\prod_i P_X(x_i)\right) e^{\gamma(t)^2\langle \mathbf{x},\mathbf{x}_0\rangle + \gamma(t)\langle \mathbf{z},\mathbf{x}\rangle - \frac{\gamma(t)^2}{2}\|\mathbf{x}\|^2} \left(\prod_{i<j} e^{\frac{1}{\Delta\sqrt{N}}\tilde{z}_{ij}x_i x_j + \frac{1}{\Delta \mathrm{N}} x_i x_j [\mathbf{x}_0]_i [\mathbf{x}_0]_j - \frac{1}{2\Delta N} x_i^2 x_j^2}\right) \tag{9}$$

With respect to the original model, the tilted measure simply includes in addition, a field in the planted direction, a random field and a renormalization of the constant in front of the quadratic part. As mentioned in Appendix 1, this corresponds to an equivalent inference problem with an additional Gaussian measurement.

The **pinned measure** is slightly different. In this case, it modifies the original problems as (denoting the pinned list as S_θ):

$$P_\theta(\mathbf{x}) \;=\; \frac{1}{Z_\theta}\left(\prod_{i\notin S_\theta} P_X(x_i)\right)\left(\prod_{i\in S_\theta}\delta(x_i - x_i^*)\right)\left(\prod_{i<j} e^{\frac{1}{\Delta\sqrt{N}}\tilde{z}_{ij}x_i x_j + \frac{1}{\Delta \mathrm{N}} x_i x_j [\mathbf{x}_0]_i [\mathbf{x}_0]_j - \frac{1}{2\Delta N} x_i^2 x_j^2}\right) \tag{10}$$

Let us now present the expressions for the replica potential, the AMP algorithm and the State Evolution equations for the sparse rank-one matrix factorization problem. For the details of the derivations, we refer the reader to (23), since the technique used is the same. Nevertheless, as far as we know, the expressions presented in the following for the tilted and the pinning measures are new and were not presented in this form in previous works.

A.1. Replica free entropy. The replica formula for such problems can be found in many places and we refer to the mathematical literature for the detailed rigorous statements: (18, 25–27, 35, 37). In particular, (18) gives a generic proof using the adaptive interpolation methods in the presence of a Gaussian channel is added, which turns out to give the same measure as the tilted one, while (27) uses instead a pinned measure. In both case, we can thus adapt the results in the literature: The asymptotic free entropy is given by the maximum of the so-called replica symmetric potential(29) :

$$\frac{1}{N}\mathbb{E}_{\mathbf{x}_0,\mathbf{z},\tilde{\mathbf{z}}} \log Z_\gamma \;\xrightarrow[N\to\infty]{}\; \operatorname{argmax}\, \Phi_{RS}(m) \tag{11}$$

$$\Phi_{RS}(m) \;=\; \mathbb{E}_{w,x_0}\left[\log Z_x\left(\frac{m}{\Delta}, \frac{m}{\Delta}x_0 + \sqrt{\frac{m}{\Delta}}w\right)\right] - \frac{m^2}{4\Delta} \tag{12}$$

where m is the order parameter of the problem, $x_0 \sim P_X$, $w \sim \mathcal{N}(0,1)$ while Z_x depends on the specific measure considered. The same is valid for the pinned measure, as long as one substitutes Z_γ with Z_θ.

Tilted measure: In the case of the **tilted measure** in Eq. [1], we have

$$\begin{aligned} Z_x(A,B;x_0) &= \int \mathrm{d}x P_X(x)\exp(\gamma^2 x x_0 + \gamma w x - \gamma^2 x^2/2)\exp(Bx - Ax^2/2) \\ &= \rho e^{-(A+\gamma^2)/2}\cosh(B + \gamma w + \gamma^2 x_0) + (1-\rho) \end{aligned} \tag{13}$$

that we can put into Eq. [12] to get

$$\begin{aligned} \Phi_{\mathrm{RS}}(\chi) =& \rho\mathbb{E}_w\left[\log\left((1-\rho) + \rho e^{-\widetilde{\chi}/2}\cosh\left(\widetilde{\chi} + \sqrt{\widetilde{\chi}}w\right)\right)\right] \\ &+ (1-\rho)\mathbb{E}_w\left[\log\left((1-\rho) + \rho e^{-\widetilde{\chi}/2}\cosh\left(\sqrt{\widetilde{\chi}}w\right)\right)\right] - \frac{\chi^2}{4\Delta}\,, \quad \widetilde{\chi} = \frac{\chi}{\Delta} + \gamma^2\,. \end{aligned} \tag{14}$$

Pinning measure: Meanwhile, considering the **pinning measure** in Eq. [2] leads to

$$
\begin{aligned}
Z_x(A,B;x_0) &= \begin{cases} \int \mathrm{d}x P_X(x)\delta_{x,x_0}\exp(Bx - Ax^2/2) & \text{with probability } \theta \\ \int \mathrm{d}x P_X(x)\exp(Bx - Ax^2/2) & \text{with probability } 1-\theta \end{cases} \\
&= \begin{cases} P_X(x_0)\exp\left(Bx_0 - Ax_0^2/2\right) & \text{with probability } \theta \\ \rho\exp(-A/2)\cosh(B) + (1-\rho) & \text{with probability } 1-\theta \end{cases}
\end{aligned} \tag{15}
$$

which in turn gives

$$
\begin{aligned}
\Phi_{\mathrm{RS}}(\chi) =& \theta\left(\rho\log\rho + (1-\rho)\log(1-\rho) + \frac{\rho}{2}\widetilde{\chi}\right) \\
&+ (1-\theta)\Bigg[\rho\mathbb{E}_w\left[\log\left((1-\rho) + \rho e^{-\widetilde{\chi}/2}\cosh\left(\widetilde{\chi} + \sqrt{\widetilde{\chi}}w\right)\right)\right] \\
&+ (1-\rho)\mathbb{E}_w\left[\log\left((1-\rho) + \rho e^{-\widetilde{\chi}/2}\cosh\left(\sqrt{\widetilde{\chi}}w\right)\right)\right]\Bigg] - \frac{\chi^2}{4\Delta}, \quad \widetilde{\chi} = \frac{\chi}{\Delta}.
\end{aligned} \tag{16}
$$

A.2. Message-Passing algorithm. The derivation of the AMP algorithm for this problem has a long history, and is connected to the Thouless-Anderson-Palmer (TAP) equations (38). For this problem, the introduction of TAP as an iterative algorithm is due to Bolthausen (39) and has been adapted to the present situation in (25, 34).

Tilted measure: The equivalence between the tilted and the planted measure with external field allows us to reduce the AMP iterations for the tilted measure to the ones for an associated inference problem. In (23), the authors provided a framework for deriving the AMP iterates for such an inference problem involving pair-wise interactions between spins. While the tilted measure defined by Eq. [9] involves additional random and planted fields, the generality of the derivation in (23) allows them to be straightforwardly incorporated into the single-site factors $P_X(x_i)$ in (23). Through an adaptation of the derivation of Equations 66, 67 in (23), we obtain:

$$
\begin{cases}
\widehat{x}_i^{t+1} = \dfrac{\rho\tanh(B_i^t + \frac{\alpha(t)}{\beta(t)^2}[\mathbf{Y}_t]_i)}{\rho + \frac{(1-\rho)\exp((A^t+\gamma^2)/2)}{\cosh(B_i^t + \frac{\alpha(t)}{\beta(t)^2}[\mathbf{Y}_t]_i)}}, \quad \sigma_i^{t+1} = \rho\dfrac{\rho + (1-\rho)e^{(A^t+\gamma^2)/2}\cosh(B_i^t + \frac{\alpha(t)}{\beta(t)^2}[\mathbf{Y}_t]_i)}{\left(\rho\cosh(B_i^t + \frac{\alpha(t)}{\beta(t)^2}[\mathbf{Y}_t]_i) + (1-\rho)\exp((A+\gamma^2)/2)\right)^2} \\
A^t = \frac{\|\widehat{\mathbf{x}}^{\mathbf{t}}\|_2^2}{\Delta N}; \quad B_i^t = \frac{1}{\Delta\sqrt{N}}\mathbf{J_i}\cdot\widehat{\mathbf{x}}^{\mathbf{t}} - \frac{1}{N\Delta}\widehat{x}_i^{t-1}\sum_k \sigma_k^t
\end{cases} \tag{17}
$$

where $\alpha(t)$ and $\beta(t)$ are the functions defining the interpolant process, fixed at the start, and $\mathbf{Y}_t$ is the value of the noisy observation at time t.

Pinning measure: When considering the pinning measure in Eq. [2], the AMP equations are only a slight variation of the ones presented for the flow-based case.

Specifically, in autoregressive-based sampling we choose a fraction θ of the variables, for which we fix $\widehat{x}_i^t = [\mathbf{x}_0]_i$, $\sigma_i^t = 0$; this is due to the fact that their posterior means are completely polarized on the solution. For the rest of the variables, a fraction $1-\theta$, the AMP equations are exactly the ones for diffusion in Eq. [17], at $\gamma = 0$.

The resulting algorithm is thus

$$
\begin{cases}
\widehat{x}_i^{t+1} = \begin{cases} [\mathbf{x}_0]_i & \text{if } i \in S_\theta \\ \dfrac{\rho\tanh(B_i^t)}{\rho + \frac{(1-\rho)\exp(A^t/2)}{\cosh(B_i^t)}}, & \text{otherwise} \end{cases}, \quad \sigma_i^{t+1} = \begin{cases} 0 & \text{if } i \in S_\theta \\ \rho\dfrac{\rho + (1-\rho)e^{A^t/2}\cosh(B_i^t)}{\left(\rho\cosh(B_i^t) + (1-\rho)\exp(A/2)\right)^2} & \text{otherwise} \end{cases} \\
A^t = \frac{\|\widehat{\mathbf{x}}^{\mathbf{t}}\|_2^2}{\Delta N}; \quad B_i^t = \frac{1}{\Delta\sqrt{N}}\mathbf{J_i}\cdot\widehat{\mathbf{x}}^{\mathbf{t}} - \frac{1}{N\Delta}\widehat{x}_i^{t-1}\sum_k \sigma_k^t
\end{cases} \tag{18}
$$

A.3. State Evolution equations. The advantage of AMP is that it can be rigorously tracked by the State Evolution equations (28, 34, 39) that turn out to be nothing but the fixed point equations of the associated replica free entropy. Again, we can use the generic results reported in (23) to get

$$
m^{t+1} = \mathbb{E}_{x_0,w}\left[f_{\mathrm{in}}\left(\frac{m^t}{\Delta}, \frac{m^t}{\Delta}x_0 + \sqrt{\frac{m^t}{\Delta}}w\right)x_0\right] \tag{19}
$$

where $x_0 \sim P_X$, $w \sim \mathcal{N}(0,1)$ and f_{in} is the input channel and depends on the specific problem.

Tilted measure: For the **tilted measure** in Eq. [1] we get

$$
\begin{aligned}
f_{\mathrm{in}}(A,B;x_0) &= \frac{\int \mathrm{d}x x P_X(x)\exp(\gamma^2 x x_0 + \gamma w x - \gamma^2 x^2/2)\exp(Bx - Ax^2/2)}{\int \mathrm{d}x P_X(x)\exp(\gamma^2 x x_0 + \gamma w x - \gamma^2 x^2/2)\exp(Bx - Ax^2/2)} \\
&= \frac{\rho\tanh(B + \gamma w + \gamma^2 x_0)}{\rho + \frac{(1-\rho)\exp\left((A+\gamma^2)/2\right)}{\cosh(B+\gamma w+\gamma^2 x_0)}}
\end{aligned} \tag{20}
$$

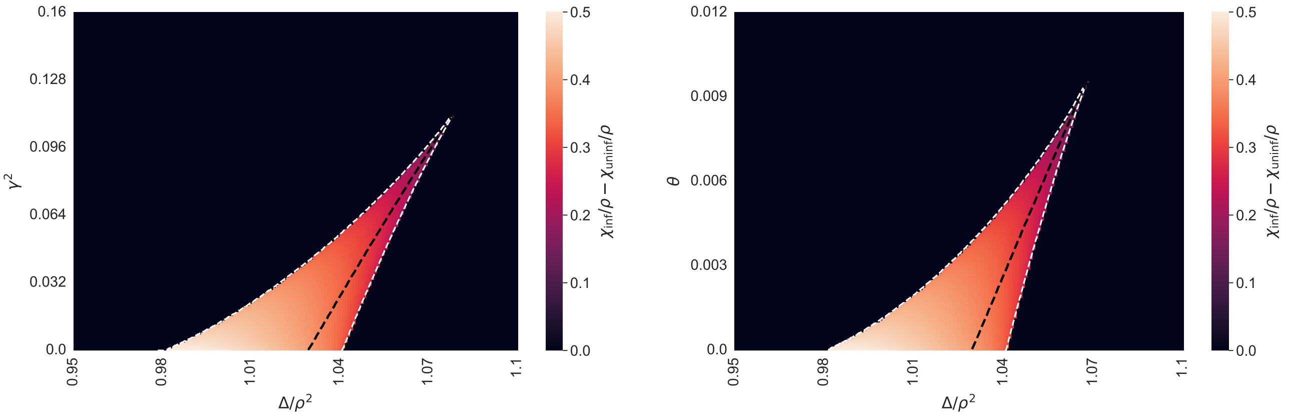

Fig. S4. Phase diagrams for flow-based sampling (left) and autoregressive-based sampling (right) for the *sparse rank-one* model, with Rademacher-Bernoulli prior and sparsity $\rho = 0.08$. On the x-axis we put the rescaled signal-to-noise-ratio Δ/ρ^2 and on the y-axis the ratio $\gamma^2 = \alpha^2/\beta^2$ (left) and the decimated ratio θ (right). We compute the order parameter χ/ρ, defined in Eq. [7], both from an uninformed and an informed initialization, and we plot the difference between the two. The dashed white lines are the *spinodal lines*, while the dashed black one is the *IT threshold*. Note that for the flow-based case (left panel), we show explicitly in Fig. S5 and Fig. S6 the behaviour of the free entropy functional for $\Delta/\rho^2 = 1.05$ and $\Delta/\rho^2 = 0.98$. Here in both plots (left and right) we have that the dynamical transition is at $\Delta_d/\rho^2 \approx 1.041$, the IT/Kauzmann transition is at $\Delta_{IT}/\rho^2 \approx 1.029$, while the tri-critical points are at $\Delta_{\rm tri}/\rho^2 \approx 1.08$ for flow-based and $\Delta_{\rm tri}/\rho^2 \approx 1.069$ for autoregressive based sampling.

which leads to the state evolution equations:

$$\chi^{t+1} = \rho^2 \mathbb{E}_w \left[\frac{\tanh(\widetilde{\chi}^t + \sqrt{\widetilde{\chi}^t} w)}{\rho + \frac{(1-\rho)\exp(\widetilde{\chi}^t/2)}{\cosh(\widetilde{\chi}^t + \sqrt{\widetilde{\chi}^t} w)}} \right], \quad \widetilde{\chi}^t \equiv \frac{\chi^t}{\Delta} + \gamma^2, \quad w \sim \mathcal{N}(0,1) \qquad [21]$$

Pinning measure: In the same way, the **pinning measure** in Eq. [2] is associated to

$$f_{\rm in}(A, B; x_0) = \begin{cases} \frac{P_X(x_0) x_0 \exp\left(Bx_0 - Ax_0^2/2\right)}{P_X(x_0)\exp\left(Bx_0 - Ax_0^2/2\right)} & \text{with probability } \theta \\ \frac{\int {\rm d}x x P_X(x) \exp(Bx - Ax^2/2)}{\int {\rm d}x P_X(x)\exp(Bx - Ax^2/2)} & \text{with probability } 1-\theta \end{cases}$$
$$= \begin{cases} x_0 & \text{with probability } \theta \\ \rho \tanh(B)/(\rho + (1-\rho)\exp(A/2)/\cosh(B)) & \text{with probability } 1-\theta \end{cases} \qquad [22]$$

and consequently to the fixed point equations

$$\frac{\chi^{t+1}}{\rho} = \theta + (1-\theta)\mathbb{E}_z \left[\frac{\rho \tanh(\widetilde{\chi}^t + \sqrt{\widetilde{\chi}^t} w)}{\rho + \frac{(1-\rho)\exp(\widetilde{\chi}^t/2)}{\cosh(\widetilde{\chi}^t + \sqrt{\widetilde{\chi}^t} w)}} \right], \quad \widetilde{\chi}^t \equiv \frac{\chi^t}{\Delta}, \quad w \sim \mathcal{N}(0,1)\,. \qquad [23]$$

A.4. Phase diagrams. In Fig. S4 we present the phase diagrams for the sparse rank-one matrix factorization problem, choosing $\rho = 0.08$ as value for sparsity. We remind that ρ must be small enough to observe a first-order phenomenology (35).

The section of the parameter space displayed is the same as in the plots presented in the main text, but here we display directly the difference $\chi_{\rm inf} - \chi_{\rm uninf}$, so that the coloured zones of the plots are the ones displaying multiple fixed points, as opposed to the black ones. We furthermore draw as white dashed lines the spinodal points, and as a black dashed line the IT threshold, both defined at the beginning of Appendix 3. For the flow-based plot, we also show explicitly in Fig. S5 and Fig. S6 the behaviour of the free entropy functional for $\Delta/\rho^2 = 1.05$ and $\Delta/\rho^2 = 0.98$. For Fig. S5 the first order transition is apparent, while for Fig. S6 the transition is found to be continuous.

Autoregressive networks vs Flows As we can see from the plots, for this model the tri-critical point for flow-based sampling is at $\Delta_{\rm tri}/\rho^2 \approx 1.08$, while for autoregressive-based sampling is at $\Delta_{\rm tri}/\rho^2 \approx 1.069$, meaning that the gap with MCMC and Langevin sampling is smaller in this latter case. In other words, this means that there is a range of values of inverse-SNR Δ for which autoregressive networks based sampling (along with MCMC and Langevin) is efficient, while flow-based sampling is not.

This appears not to be specific to this particular value of ρ, but in all the range of sparsity in which the model presents a first order phase transition, autoregressive-based sampling appears to be more efficient than flow-based sampling, in the sense explained above, as shown in Fig. S7.

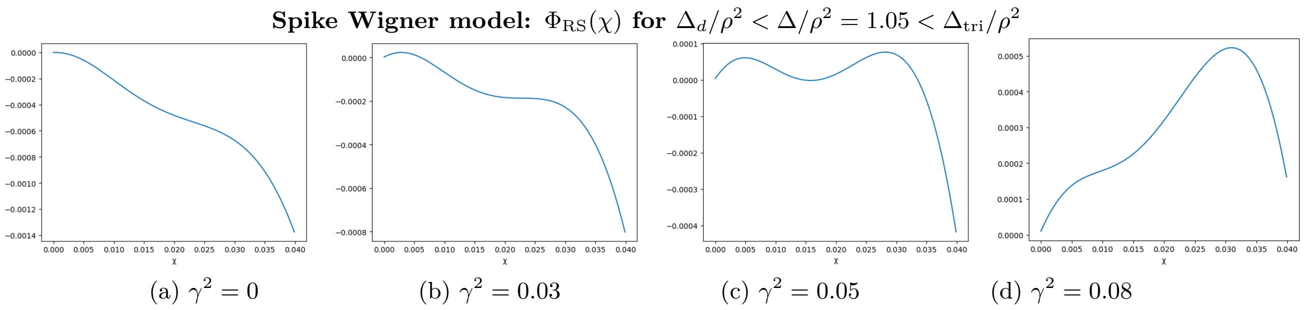

Fig. S5. The free entropy function $\Phi_{\rm RS}(\chi)$ in the region where the flow-based model fails because of the jump around panel(c).

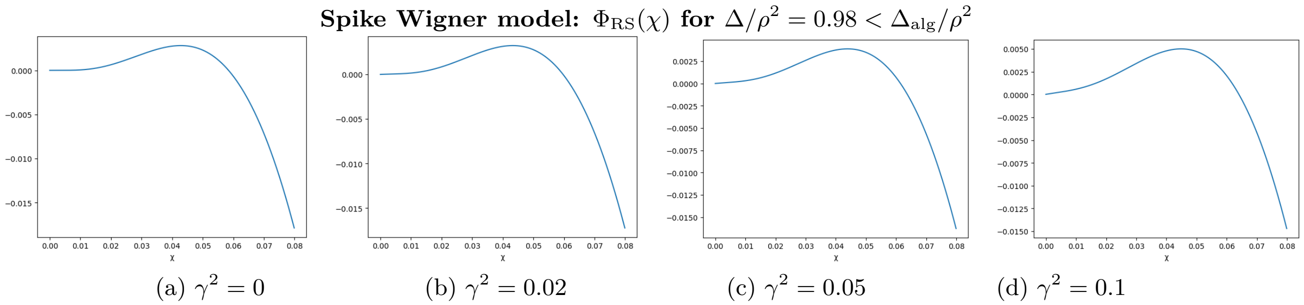

Fig. S6. The free entropy function $\Phi_{\rm RS}(\chi)$ in the region where the flow-based model succeeds as the position of the maxima is always unique.

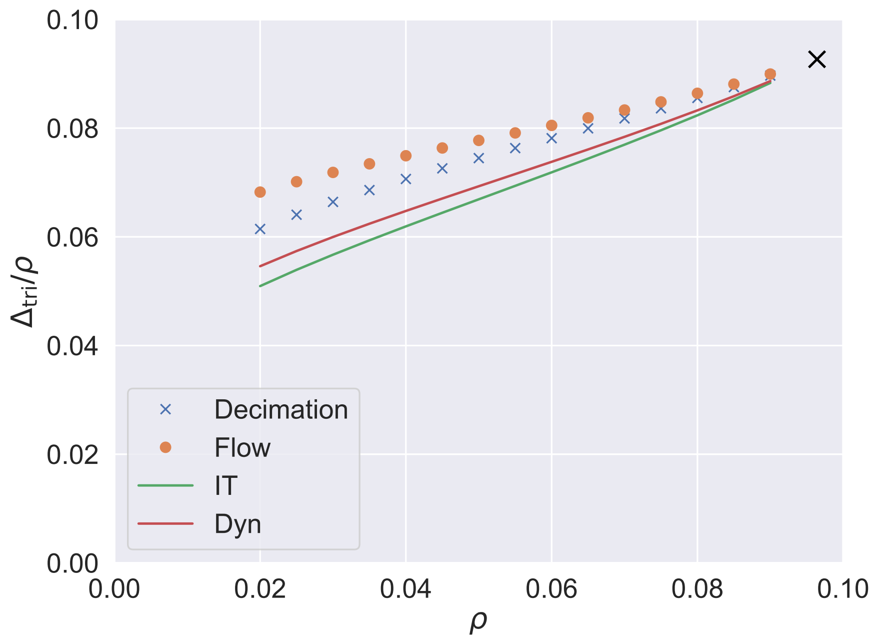

Fig. S7. Flows vs autoregressive in the sparse rank-one problem. We plot the values of the tri-critical point for flow-based (orange dots) and autoregressive-based (blue crosses) sampling on the sparse rank-one model, when varying the sparsity parameter ρ. Specifically, we plot $\Delta_{\rm tri}/\rho$ for the two cases, comparing them also to the Dynamical transition value Δ_d/ρ (red line) and the IT transition value $\Delta_{\rm IT}/\rho$ (green line). Finally, we also put a black cross at the point $(\rho_{\max}, \Delta_{\max}/\rho_{\max})$, taken from (23), which corresponds to the maximum value of ρ at which there is a first order phase transition.

The easy phase (and a subtle point) For the expert reader, it is worth pointing a subtle difference between what happens at $\Delta = \rho^2$ and $\Delta = \Delta_{\rm alg} < \rho^2$ (see also (23)). Indeed, we use here the definition of the easy phase as the absence of a metastable maxima that is trapping the dynamics. In other words, the state evolution of AMP initialized at the uninformed fixed point

finds the global maxima. This is indeed what is going on for $\Delta = \Delta_{\rm alg}$, as illustrated in Figure S6.

However, it is worth mentioning that already below $\Delta = \rho^2$, the fixed point at zero is unstable, so that there are two fixed point: the "correct" one at large χ, and the one found by AMP at low but non-zero χ. This phenomenon, usually called the Baik-Ben Arous-Peche (BBP) transition, is illustrated in Figure S8. In this phase, while AMP, for instance (but also a standard spectral method such as PCA (23, 40)) would find an estimator correlated with the ground truth (and thus $\chi^* > 0$ even at $\gamma = 0$), there is still a first-order phase transition and AMP would not be optimal. Since there is a discontinuity, the flow-based method suffers from the same problem, and fails to sample as well.

The BBP transition at $\gamma^2 = 0$: $\Phi_{\rm RS}(\chi)$ for $\Delta/\rho^2 = 0.99$ (left) and $\Delta/\rho^2 = 1.01$ (right)

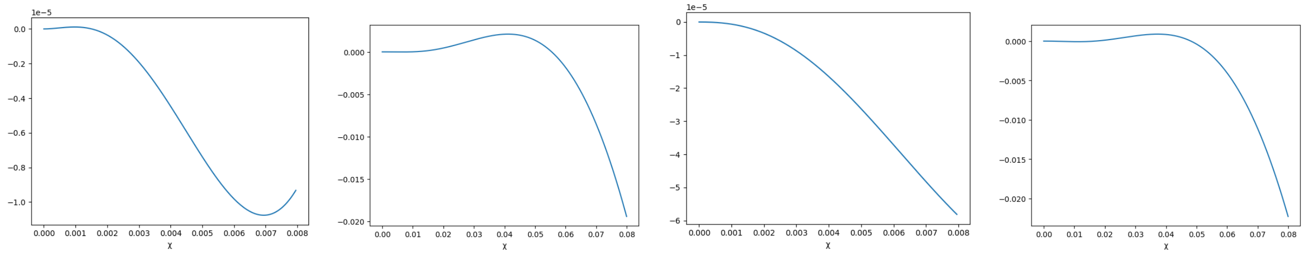

(a) $\Delta/\rho^2 = 0.99$ (zoom) (b) $\Delta/\rho^2 = 0.99$ (unzoom) (c) $\Delta/\rho^2 = 1.01$ (zoom) (d) $\Delta/\rho^2 = 1.01$ (unzoom)

Fig. S8. Illustration of the BBP(40) transition around $\Delta = \rho^2$ where the spurious minima stop to be at zero (see the difference between the zoomed (a) and (c)), but there the correct non-spurious minima is still at larger value of χ (see the unzoomed (b) and (d)). This is still a hard phase for inference and sampling, and the problem remains so until $\Delta < \Delta_{\rm alg}$

B. Ising p-spin model. We consider now the p-spin model (here with $p = 3$), which is one of the most important statistical physics problems in spin glass theory. First, let us look at the Ising version, defined by the following Hamiltonian:

$$\mathcal{H}(\mathbf{x}) = -\frac{\sqrt{3}}{N} \sum_{i<j<k} J_{ijk} x_i x_j x_k \,, \qquad [24]$$

where $J_{ijk} \sim \mathcal{N}(0, 1)$ and the variables x_i are constrained to be ± 1. This version of the model was introduced in (41), and solved with the replica method in (42). It is hard to underestimate its importance in the spin glass and glass theory as the prototype of the mean-field random first-order model (43–47).

For temperatures higher than the spin glass, or Kauzmann, temperature, the model can be proven (3–5) to be contiguous to its “planted version", the tensor factorization problem (7). This is nothing but the tensor generalization of the former matrix estimation problem of the preceding section. We shall report the derivations presented in (4), which considers the planted version of the problem, but that (thanks to contiguity) also describe the unplanted model, and the mapping between the two can be done using $\Delta = \frac{2}{3}T^2$.

As before, from a Bayesian perspective, the problem boils down to sampling the following posterior

$$P_0(\mathbf{x}) \propto \prod_i P_X(x_i) \prod_{i<j<k} e^{\frac{\sqrt{3}\beta}{N} J_{ijk} x_i x_j x_k} \,, \qquad [25]$$

where the prior $P_X(x) = \delta_{x,-1}/2 + \delta_{x,+1}/2$ constrains the variables to be ± 1.

As we have reminded for the previous model, when considering the tilted measure in Eq. [1] we will exploit again the fact that considering an equilibrium configuration $\mathbf{x}_0$ is equivalent to take as $\mathbf{x}_0$ the planted configuration, as long as we are beyond the Spin Glass temperature.

We shall thus consider the following **tilted measure** for diffusion and flow-based sampling:

$$P_\gamma(\mathbf{x}) = \frac{1}{Z_\gamma} \left(\prod_i P_X(x_i) \right) e^{\gamma(t)^2 \langle \mathbf{x}, \mathbf{x}_0 \rangle + \gamma(t) \langle z, \mathbf{x} \rangle - \frac{\gamma(t)^2}{2} \|\mathbf{x}\|^2} \prod_{i<j<k} e^{\frac{\sqrt{3}\beta}{N} J_{ijk} x_i x_j x_k} \qquad [26]$$

As before, we can notice that with respect to the original measure in Eq. [25], the tilting adds a field in the planted direction, a random field and a normalization factor depending on the l2 norm.

For the **pinned measure** defined in Eq. [2] the original problem becomes (denoting as S_θ the pinned list):

$$P_\theta(\mathbf{x}) = \frac{1}{Z_\theta} \left(\prod_{i \notin S_\theta} P_X(x_i) \right) \left(\prod_{i \in S_\theta} \delta(x_i - x_i^*) \right) \prod_{i<j<k} e^{\frac{\sqrt{3}\beta}{N} J_{ijk} x_i x_j x_k} \qquad [27]$$

Let us now present the expressions for the replica potential, the AMP algorithm and the State Evolution equations for the Ising p-spin model. For the details of the derivations, we refer the reader to (4), since the technique used is the same. Nevertheless, as far as we know, the expressions presented in the following for the tilted and the pinning measures are new and were not presented in this form in previous works.

B.1. Replica free entropy. The replica formula for this model has been studied extensively in the literature, and can also be proven rigorously, see (4). Again, the asymptotic free entropy is given by the maximum of the so-called replica symmetric potential (29) :

$$\frac{1}{N}\mathbb{E}_{\mathbf{x}_0,\mathbf{z},J}\log Z_\gamma \xrightarrow[N\to\infty]{} \text{argmax}\ \Phi_{RS}(m) \tag{28}$$
$$\Phi_{RS}(m) = \mathbb{E}_{w,x_0}\left[\log Z_x\left(\frac{m^2}{\Delta},\frac{m^2}{\Delta}x_0+\sqrt{\frac{m^2}{\Delta}}w\right)\right]-\frac{m^3}{3\Delta} \tag{29}$$

where m is the order parameter of the problem, $x_0\sim P_X$, $w\sim\mathcal{N}(0,1)$ while Z_x depends on the specific measure considered. The same is valid for the pinned measure, as long as one substitutes Z_γ with Z_θ.

In the following, since we will be interested on the unplanted model, we will use the temperature $T=\sqrt{3\Delta/2}$ as signal-to-noise parameter.

Tilted measure: In the case of the **tilted measure** in Eq. [1] we have

$$\begin{aligned} Z_x(A,B;x_0) &= \int \mathrm{d}x P_X(x)\exp(\gamma^2 x x_0+\gamma w x-\gamma^2x^2/2)\exp(Bx-Ax^2/2)\\ &= e^{-(A+\gamma^2)/2}\cosh(B+\gamma w+\gamma^2 x_0)\end{aligned} \tag{30}$$

that can be put into Eq. [28] to get

$$\Phi_{\mathrm{RS}}(\chi) = -\frac{\widetilde{\chi}}{2}+\mathbb{E}_w\left[\log\cosh\left(\widetilde{\chi}+\sqrt{\widetilde{\chi}}w\right)\right]-\frac{\chi^3}{2T^2}\,,\quad \widetilde{\chi}=\frac{3\chi^2}{2T^2}+\gamma^2\,. \tag{31}$$

Pinning measure: Meanwhile, considering the **pinning measure** in Eq. [2] leads to

$$\begin{aligned} Z_x(A,B;x_0) &= \begin{cases}\int \mathrm{d}x P_X(x)\delta_{x,x_0}\exp(Bx-Ax^2/2) & \text{with probability } \theta\\ \int \mathrm{d}x P_X(x)\exp(Bx-Ax^2/2) & \text{with probability } 1-\theta\end{cases}\\ &= \begin{cases}P_X(x_0)\exp\left(Bx_0-Ax_0^2/2\right) & \text{with probability } \theta\\ \exp(-A/2)\cosh(B) & \text{with probability } 1-\theta\end{cases}\end{aligned} \tag{32}$$

which in turn gives

$$\Phi_{\mathrm{RS}}(\chi) = \frac{2\theta-1}{2}\widetilde{\chi}+(1-\theta)\mathbb{E}_w\left[\log\cosh\left(\widetilde{\chi}+\sqrt{\widetilde{\chi}}w\right)\right]-\frac{\chi^3}{2T^2}\,,\quad \widetilde{\chi}=\frac{3\chi^2}{2T^2}\,. \tag{33}$$

B.2. Message-passing algorithm. As for the SK model and the sparse rank-one matrix factorization problem, the AMP iterates and the associated Thouless-Anderson-Palmer (TAP) equations (38) have been widely studied for the p-spin models (48). Here we consider the formalism of (4), which presents the AMP iterates for the Spike Tensor model, and we use contiguity to derive equations valid for the unplanted model with the tilting (or pinning) field. We remind again that the mapping between the two models is given by $\Delta=\frac{2T^2}{3}$.

Tilted measure: Due to contiguity with the planted model and the equivalence of the tilted measure and an associated measure with a planted field described in Section 1, the AMP iterations for the tilted measure can be obtained through the ones for the associated inference problem described in (23).This yields to

$$\begin{cases}\widehat{x}_i^{t+1}=\tanh(B_i^t+\frac{\alpha(t)}{\beta(t)^2}[\mathbf{Y}_t]_i), \quad \sigma_i^{t+1}=\cosh(B_i^t+\frac{\alpha(t)}{\beta(t)^2}[\mathbf{Y}_t]_i)^{-2}\\ B_i^t=\frac{\sqrt{3}\beta}{N}\sum_{j<k}J_{ijk}\widehat{x}_j^t\widehat{x}_k^t-\frac{3}{N}\beta^2\widehat{x}_i^{t-1}\widehat{\mathbf{x}}^t\cdot\widehat{\mathbf{x}}^{t-1}\sum_k\sigma_k^t/N\end{cases} \tag{34}$$

where $\alpha(t)$ and $\beta(t)$ are the functions defining the interpolant process, fixed at the start, and $\mathbf{Y}_t$ is the value of the noisy observation at time t.

Pinning measure: The AMP equations for the pinning measure in Eq. [2] are a slight variation of the ones just presented for the tilted measure.

Specifically, in the autoregressive-based sampling scheme for a fraction θ of the variables we fix $\widehat{x}_i^t=x_0$, $\sigma_i^t=0$, since their posterior means are totally polarized on the solution. For the rest of the variables, a fraction $1-\theta$, the AMP equations are the same as the one presented for diffusion in Eq. [34], provided that we fix $\gamma=0$.

The resulting equations are:

$$\begin{cases}\widehat{x}_i^{t+1}=\begin{cases}[\mathbf{x}_0]_i & \text{if } i\in S_\theta\\ \tanh(B_i^t), & \text{otherwise}\end{cases}, \quad \sigma_i^{t+1}=\begin{cases}0 & \text{if } i\in S_\theta\\ \cosh(B_i^t)^{-2} & \text{otherwise}\end{cases}\\ B_i^t=\frac{\sqrt{3}\beta}{N}\sum_{j<k}J_{ijk}\widehat{x}_j^t\widehat{x}_k^t-\frac{3}{N}\beta^2\widehat{x}_i^{t-1}\widehat{\mathbf{x}}^t\cdot\widehat{\mathbf{x}}^{t-1}\sum_k\sigma_k^t/N\end{cases} \tag{35}$$

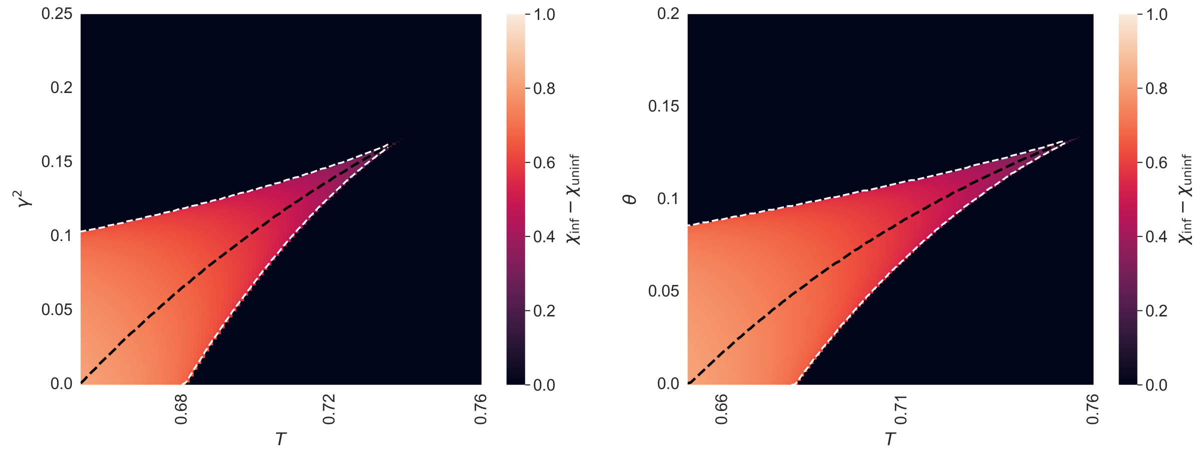

Fig. S9. Phase diagrams for flow-based sampling (left) and autoregressive-based sampling (right) for the *Ising p-spin* model with $p = 3$. On the x-axis we put the temperature T and on the y-axis the ratio $\gamma^2 = \alpha^2/\beta^2$ (left) and the decimated ratio θ (right). We compute the order parameter χ, defined in Eq. [7], both from an uninformed and an informed initialization, and we plot the difference between the two. The dashed white lines are the *spinodal lines*, while the dashed black one is the *IT threshold*, both defined at the beginning of the section. In both plots we have that the dynamical transition is at $T_d \approx 0.682$, the Kauzmann transition is at $T_K \approx 0.652$, while the tri-critical points are at $T_{\rm tri} \approx 0.741$ for flow-based and $T_{\rm tri} \approx 0.759$ for autoregressive-based sampling.

B.3. State evolution equations. The advantage of AMP is that it can be rigorously tracked by the State Evolution equations (28), that can be proven to be nothing but the fixed point of the replica potential in Eq. [28]. We can use the formalism in (4) to get

$$m^{t+1} = \mathbb{E}_{x_0,w}\left[f_{\rm in}\left(\frac{(m^t)^2}{\Delta}, \frac{(m^t)^2}{\Delta}x_0 + \sqrt{\frac{(m^t)^2}{\Delta}}w\right)x_0\right] \quad [36]$$

where $x_0 \sim P_X$, $w \sim \mathcal{N}(0,1)$ and f_{in} is the input channel and depends on the specific problem. Again, we will state our results using the temperature $T = \sqrt{3\Delta/2}$.

Tilted measure: For the **tilted measure** in Eq. [1] we have

$$\begin{aligned} f_{\rm in}(A,B;x_0) &= \frac{\int {\rm d}x x P_X(x)\exp(\gamma^2 x x_0 + \gamma w x - \gamma^2 x^2/2)\exp(Bx - Ax^2/2)}{\int {\rm d}x P_X(x)\exp(\gamma^2 x x_0 + \gamma w x - \gamma^2 x^2/2)\exp(Bx - Ax^2/2)} \\ &= \tanh(B + \gamma w + \gamma^2 x_0) \end{aligned} \quad [37]$$

which leads to the State Evolution equations

$$\chi^{t+1} = \mathbb{E}_w\left[\tanh(\widetilde{\chi}^t + \sqrt{\widetilde{\chi}^t}w)\right], \quad \widetilde{\chi}^t \equiv \frac{3(\chi^t)^2}{2T^2} + \gamma^2, \quad w \sim \mathcal{N}(0,1) \quad [38]$$

Pinning measure: In the same way, for the **pinning measure** in Eq. [2] we get

$$\begin{aligned} f_{\rm in}(A,B;x_0) &= \begin{cases} \frac{P_X(x_0)x_0\exp\left(Bx_0 - Ax_0^2/2\right)}{P_X(x_0)\exp\left(Bx_0 - Ax_0^2/2\right)} & \text{with probability } \theta \\ \frac{\int {\rm d}x x P_X(x)\exp(Bx - Ax^2/2)}{\int {\rm d}x P_X(x)\exp(Bx - Ax^2/2)} & \text{with probability } 1-\theta \end{cases} \\ &= \begin{cases} x_0 & \text{with probability } \theta \\ \tanh(B) & \text{with probability } 1-\theta \end{cases} \end{aligned} \quad [39]$$

and thus the fixed point equations

$$\chi^{t+1} = \theta + (1-\theta)\mathbb{E}_w\left[\tanh(\widetilde{\chi}^t + \sqrt{\widetilde{\chi}^t}w)\right], \quad \widetilde{\chi}^t \equiv \frac{3(\chi^t)^2}{2T^2}, \quad w \sim \mathcal{N}(0,1) \quad [40]$$

B.4. Phase diagrams. The phase diagrams for the Ising p-spin are presented in Figure S9, in the same style as the previous section. In this case, notably, we observe that the flow-based method is advantageous with respect to the autoregressive one, in the sense that the former shows a smaller gap $T_{\rm tri} - T_d$ compared to the latter. This is the opposite situation compared to the previous problem.

C. Spherical p-spin model. The spherical p-spin model (here we will consider the case $p = 3$ for simplicity) is a variation of the Ising model where the configurations are constrained to lie on the sphere $\mathbf{x} \in \mathcal{S}^{N-1}$ (48). Again, we shall use the planted version, which is asymptotically equivalent at high temperature with the unplanted problem having independent Gaussian entries. The Hamiltonian thus still reads:

$$\mathcal{H}(\mathbf{x}) = -\frac{\sqrt{3}}{N} \sum_{i<j<k} J_{ijk} x_i x_j x_k \,, \tag{41}$$

where $J_{ijk} \sim \mathcal{N}(0,1)$ and now $P_X(x_i) = \mathcal{N}(0,1)$.

Same as its Ising version, for temperatures higher than the Kauzmann temperature the model can be proven (4) to be contiguous to its "planted version", the tensor factorization problem (7). Here we report the derivations presented in (4), and the mapping between the two is again $\Delta = \frac{2}{3}T^2$.

From a Bayesian point of view, the posterior measure we want to sample is the following:

$$P_0(\mathbf{x}) \propto \prod_i P_X(x_i) \prod_{i<j<k} e^{\frac{\sqrt{3}\beta}{N} J_{ijk} x_i x_j x_k} \,, \tag{42}$$

where $P_X(x) = \mathcal{N}(0,1)$ in high-dimension constrains the variables to be on the sphere.

We first consider the following **tilted measure**, arising in diffusion and flow-based sampling:

$$P_\gamma(\mathbf{x}) = \frac{1}{Z_\gamma} \left(\prod_i P_X(x_i) \right) e^{\gamma(t)^2 \langle \mathbf{x}, \mathbf{x}_0 \rangle + \gamma(t) \langle z, \mathbf{x} \rangle - \frac{\gamma(t)^2}{2} \|\mathbf{x}\|^2} \prod_{i<j<k} e^{\frac{\sqrt{3}\beta}{N} J_{ijk} x_i x_j x_k} \tag{43}$$

Again, with respect to the original measure in Eq. [42], this measure presents an additional field in the planted direction, a random field and a constant factor depending on the l2 norm.

For the **pinned measure** defined in Eq. [2] the original problem becomes (denoting as S_θ the pinned list):

$$P_\theta(\mathbf{x}) = \frac{1}{Z_\theta} \left(\prod_{i \notin S_\theta} P_X(x_i) \right) \left(\prod_{i \in S_\theta} \delta(x_i - x_i^*) \right) \prod_{i<j<k} e^{\frac{\sqrt{3}\beta}{N} J_{ijk} x_i x_j x_k} \tag{44}$$

Let us now present the expressions for the replica potential, the AMP algorithm and the State Evolution equations for the Spherical p-spin model. For the details of the derivations, we refer the reader to (4), since the technique used is the same. Nevertheless, as far as we know, the expressions presented in the following for the tilted and the pinning measures are new and were not presented in this form in previous works.

C.1. Replica free entropy. The replica free entropy for this model has been studied extensively in the spin-glass literature, and can also be proven rigorously, see e.g. (4).

As for the previous models, the asymptotic free entropy is given by the maximum of the so-called replica symmetric potential (29) :

$$\frac{1}{N} \mathbb{E}_{\mathbf{x}_0, \mathbf{z}, J} \log Z_\gamma \xrightarrow[N \to \infty]{} \operatorname{argmax} \Phi_{RS}(m) \tag{45}$$

$$\Phi_{RS}(m) = \mathbb{E}_{w, x_0} \left[\log Z_x \left(\frac{m^2}{\Delta}, \frac{m^2}{\Delta} x_0 + \sqrt{\frac{m^2}{\Delta}} w \right) \right] - \frac{m^3}{3\Delta} \tag{46}$$

where m is the order parameter of the problem, $x_0 \sim P_X$, $w \sim \mathcal{N}(0,1)$ and Z_x depends on the specific measure considered. The same is valid for the pinned measure, as long as one substitutes Z_γ with Z_θ.

While using the same derivation, in the following we will use $T = \sqrt{3\Delta/2}$ as signal-to-noise parameter, since we are interested in studying the unplanted model.

Tilted measure: In the case of the **tilted measure** in Eq. [1] we have

$$\begin{aligned} Z_x(A, B; x_0) &= \int \mathrm{d}x P_X(x) \exp(\gamma^2 x x_0 + \gamma w x - \gamma^2 x^2/2) \exp(Bx - Ax^2/2) \\ &= \sqrt{\frac{2\pi}{A + \gamma^2 + 1}} \exp\left(\frac{(B + \gamma^2 x_0 + \gamma w)^2}{2(A + \gamma^2 + 1)} \right) \end{aligned} \tag{47}$$

which leads to

$$\Phi_{\rm RS}(\chi) = \frac{\widetilde{\chi}}{2} + \frac{1}{2} \log\left(\frac{2\pi}{\widetilde{\chi} + 1} \right) - \frac{1}{2T^2} \chi^3 \,, \quad \widetilde{\chi} = \frac{3\chi^2}{2T^2} + \gamma^2 \,. \tag{48}$$

Pinning measure: Meanwhile, considering the **pinning measure** in Eq. [2] leads to

$$Z_x(A,B;x_0) = \begin{cases} P_X(x_0)\exp\left(Bx_0 - \frac{A}{2}x_0^2\right) & \text{with probability } \theta \\ \sqrt{\frac{2\pi}{A+1}}\exp\left(\frac{B^2}{2(A+1)}\right) & \text{with probability } 1-\theta \end{cases} \quad [49]$$

which in turn gives

$$\Phi_{\mathrm{RS}}(\chi) = \frac{\widetilde{\chi}}{2} + \frac{1-\theta}{2}\log\left(\frac{2\pi}{\widetilde{\chi}+1}\right) - \theta\log(\sqrt{2\pi}e) - \frac{\chi^3}{2T^2}\,, \quad \widetilde{\chi} = \frac{3\chi^2}{2T^2}\,. \quad [50]$$

C.2. Message-passing algorithm. As already mentioned for the Ising version of the model, the Thouless-Anderson-Palmer (TAP) equations (38) have been widely studied for the p-spin models (48). We use the results of (4), which presents the AMP algorithm for the Spike Tensor model, and we use contiguity to derive equations valid for the unplanted model with the tilting (or pinning) field. The mapping between the two models is again given by $\Delta = \frac{2T^2}{3}$.

Tilted measure: The equivalence between the tilted and the planted measure with external field allows us to map the AMP iterations for the tilted measure to the ones for an associated inference problem. Here we consider the formalism of (4), such that the resulting equations are:

$$\begin{cases} B_i^t = \frac{\sqrt{3}\beta}{N}\sum_{j<k} J_{ijk}\widehat{x}_j^t\widehat{x}_k^t - \frac{3}{N}\beta^2\sigma^t\widehat{x}_i^{t-1}\widehat{\mathbf{x}}^t\cdot\widehat{\mathbf{x}}^{t-1} \\ \widehat{x}_i^{t+1} = \frac{B_i^t + \frac{\alpha(t)}{\beta(t)^2}[\mathbf{Y}_t]_i}{3\beta^2\|\hat{\mathbf{x}}^t\|_2^2/(2N)+\gamma^2+1}\,, \sigma^{t+1} = \frac{1}{3\beta^2\|\hat{\mathbf{x}}^t\|_2^2/(2N)+\gamma^2+1} \end{cases} \quad [51]$$

where $\alpha(t)$ and $\beta(t)$ are the functions defining the interpolant process, fixed at the start, and $\mathbf{Y}_t$ is the value of the noisy observation at time t.

Pinning measure: When considering the pinning measure in Eq. [2], the AMP equations are only a slight variation of the ones presented for the flow-based case.

Specifically, in autoregressive-based sampling we choose a fraction θ of the variables, for which we fix $\widehat{x}_i^t = x_0$, $\sigma_i^t = 0$, which stems from the fact that their posterior means are completely polarized on the solution. For the rest of the variables, a fraction $1-\theta$, the AMP equations are exactly the ones reported in Eq. [51], provided we fix $\gamma = 0$.

The resulting algorithm is the following:

$$\begin{cases} \widehat{x}_i^{t+1} = \begin{cases} [\mathbf{x}_0]_i & \text{if } i \in S_\theta \\ \frac{B_i^t}{3\beta^2\|\hat{\mathbf{x}}^t\|_2^2/(2N)+1}, & \text{otherwise} \end{cases}, \quad \sigma_i^{t+1} = \begin{cases} 0 & \text{if } i \in S_\theta \\ \frac{1}{3\beta^2\|\hat{\mathbf{x}}^t\|_2^2/(2N)+1} & \text{otherwise} \end{cases} \\ B_i^t = \frac{\sqrt{3}\beta}{N}\sum_{j<k} J_{ijk}\widehat{x}_j^t\widehat{x}_k^t - \frac{3}{N}\beta^2\widehat{x}_i^{t-1}\widehat{\mathbf{x}}^t\cdot\widehat{\mathbf{x}}^{t-1}\sum_k \sigma_k^t/N \end{cases} \quad [52]$$

C.3. State evolution equations. As mentioned earlier, AMP iterations possess the salient property of being able to be rigorously tracked by the State Evolution equations, that turn out to be the fixed point of the replica potential in Eq. [45]. Here, again we follow the results presented in (4), such that as the Ising version of the model, we have

$$m^{t+1} = \mathbb{E}_{x_0,w}\left[f_{\mathrm{in}}\left(\frac{(m^t)^2}{\Delta}, \frac{(m^t)^2}{\Delta}x_0 + \sqrt{\frac{(m^t)^2}{\Delta}}w\right)x_0\right] \quad [53]$$

where $x_0 \sim P_X$, $w \sim \mathcal{N}(0,1)$ and f_{in} is the input channel and depends on the specific problem. We also remind the mapping $T = \sqrt{3\Delta/2}$, which we will use in the following presentation.

Tilted measure: For the **tilted measure** in Eq. [1] we have

$$\begin{aligned} f_{\mathrm{in}}(A,B;x_0) &= \frac{\int \mathrm{d}x x P_X(x)\exp(\gamma^2 x x_0 + \gamma w x - \gamma^2 x^2/2)\exp(Bx - Ax^2/2)}{\int \mathrm{d}x P_X(x)\exp(\gamma^2 x x_0 + \gamma w x - \gamma^2 x^2/2)\exp(Bx - Ax^2/2)} \\ &= \frac{B + \gamma^2 x_0 + \gamma w}{A + \gamma^2 + 1} \end{aligned} \quad [54]$$

which leads to the State Evolution equations

$$\chi^{t+1} = \frac{\widetilde{\chi}^t}{1+\widetilde{\chi}^t}\,, \quad \widetilde{\chi}^t \equiv \frac{3}{2T^2}(\chi^t)^2 + \gamma^2\,. \quad [55]$$

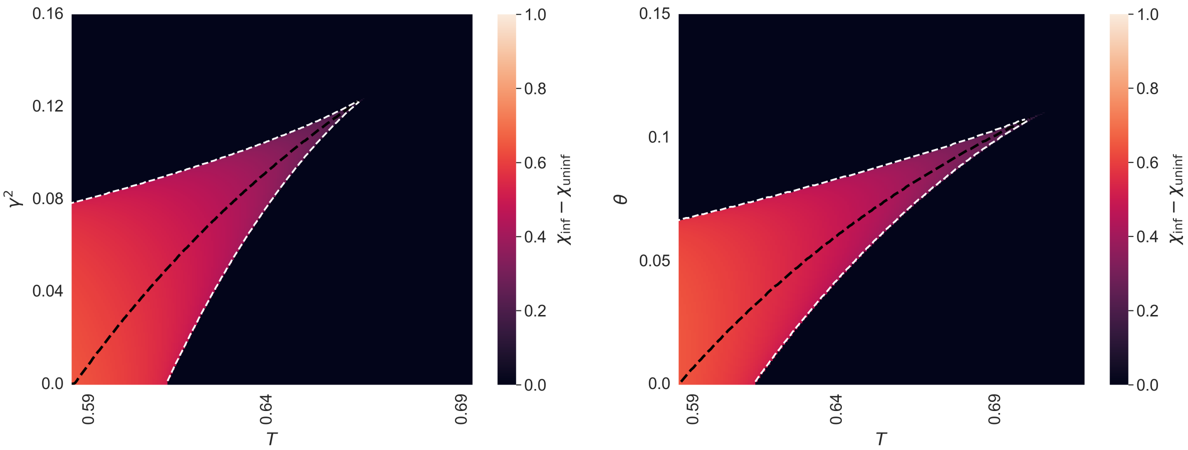

Fig. S10. Phase diagrams for flow-based sampling (left) and autoregressive-based sampling (right) for the *Spherical p-spin* model with $p = 3$. On the x-axis we put the temperature T and on the y-axis the ratio $\gamma^2 = \alpha^2/\beta^2$ (left) and the decimated ratio θ (right). We compute the order parameter χ, defined in Eq. [7], both from an uninformed and an informed initialization, and we plot the difference between the two. The dashed white lines are the *spinodal lines*, while the dashed black one is the *IT threshold*, both defined at the beginning of the section. In both plots we have that the dynamical transition is at $T_d = \sqrt{3/8}$, the Kauzmann transition is at $T_K \approx 0.58$, while the tri-critical points are at $T_{\rm tri} = 2/3$ for flow-based and $T_{\rm tri} = \sqrt{1/2}$ for autoregressive based sampling.

Pinning measure: In the same way, for the **pinning measure** in Eq. [2] we find

$$f_{\rm in}(A,B;x_0) = \begin{cases} \frac{P_X(x_0)x_0\exp\left(Bx_0 - Ax_0^2/2\right)}{P_X(x_0)\exp\left(Bx_0 - Ax_0^2/2\right)} & \text{with probability } \theta \\ \frac{\int \mathrm{d}x\, x P_X(x)\exp(Bx - Ax^2/2)}{\int \mathrm{d}x P_X(x)\exp(Bx-Ax^2/2)} & \text{with probability } 1-\theta \end{cases}$$
$$= \begin{cases} x_0 & \text{with probability } \theta \\ \frac{B}{A+1} & \text{with probability } 1-\theta \end{cases} \quad [56]$$

and thus the fixed point equations are

$$\chi^{t+1} = \theta + (1-\theta)\frac{\widetilde{\chi}^t}{1+\widetilde{\chi}^t}\,, \quad \widetilde{\chi}^t \equiv \frac{3(\chi^t)^2}{2T^2} \quad [57]$$

C.4. Phase diagrams. The phase diagrams for the spherical p-spin are presented in Figure S10, and we plot the same quantities as for the previous models. We observe again that the flow-based method is advantageous with respect to the autoregressive one.

D. The k-hypergraph bicoloring model.

D.1. Target model definition. The k-hypergraph bicoloring (or k-NAESAT) problem is a prototypical model of constrained satisfaction problems defined on hypergraphs. An instance of the problem is determined by an hypergraph $G=(V,E)$, where V is the set of N vertices and E the set of M hyperedges, each containing exactly k vertices.

Each vertex $i \in V$ is associated to an Ising-spin variable $x_i = \pm 1$, while each hyperedge $a \in E$ is associated to a constraint involving the k vertices entering in a (in the following, we will use $\mathbf{x}_{\partial a}$ to indicate this set of variables).

For bicoloring, the a-th constraint is satisfied if there is at least one $+1$ and one -1 among the k variables of $\mathbf{x}_{\partial a}$. In terms of probability distribution, this translates to

$$P_0(\mathbf{x}) = \frac{1}{Z(G)} \prod_{a=1}^{M} \omega(\mathbf{x}_{\partial a}) \,, \quad \omega(x_1,\dots,x_k) = \begin{cases} 0 & \text{if } \sum_{i=1}^{k} x_i = \pm k \\ 1 & \text{otherwise} \end{cases} \qquad [58]$$

In the following, we will focus on the asymptotic limit where both N and M go to infinity, with constant rate $\alpha = M/N$.

This model has again the advantage to be contiguous to its planted version (22). We refer to (22, 49, 50) for rigorous results, and to (30, 51–53) for results within the cavity approach.

D.2. BP equations and Bethe free entropy. To analyse the properties of the model, one can use the cavity method (54) from statistical physics, which allows to derive the BP equations for the problem (see e.g. (53)), which can be written in terms of *cavity messages* as

$$h_{i\to a} = f(\{u_{b\to i}\}_{b\in\partial_i \setminus a}) \,, \quad u_{a\to i} = g(\{h_{j\to a}\}_{j\in\partial_a\setminus i}) \qquad [59]$$

where

$$f(u_1,\dots,u_d) = \frac{\prod_{i=1}^{d}(1+u_i) - \prod_{i=1}^{d}(1-u_i)}{\prod_{i=1}^{d}(1+u_i) + \prod_{i=1}^{d}(1-u_i)} \qquad [60]$$

is the function defining the messages going from factor nodes to variable nodes and

$$g(h_1,\dots,h_{k-1}) = \frac{\sum_{x_1,\dots,x_k} \omega(x_1,\dots,x_k) x_k \prod_{i=1}^{k-1}(1+h_i x_i)}{\sum_{x_1,\dots,x_k} \omega(x_1,\dots,x_k) \prod_{i=1}^{k-1}(1+h_i x_i)} \qquad [61]$$

is the one defining messages going from variable nodes to factor nodes.

Once a fixed point of the BP equations is reached, one can compute the Free entropy from the resulting BP marginals. In its general form, it can be written as

$$\frac{1}{N}\ln Z(G) = \frac{1}{N}\sum_{i=1}^{N} \ln \mathcal{Z}_0^{\mathrm{v}}(\{u_{a\to i}\}_{a\in\partial i}) + \frac{1}{N}\sum_{a=1}^{M} \ln \mathcal{Z}_0^{\mathrm{c}}(\{h_{i\to a}\}_{i\in\partial a}) - \frac{1}{N}\sum_{(i,a)} \ln \mathcal{Z}_0^{\mathrm{e}}(h_{i\to a}, u_{a\to i}) \,, \qquad [62]$$

where the last sum runs over the edges of the factor graph, and the local partition functions are defined as:

$$\mathcal{Z}_0^{\mathrm{v}}(u_1,\dots,u_d) = \sum_x \prod_{i=1}^{d} \left(\frac{1+xu_i}{2}\right) \,, \qquad [63]$$

$$\mathcal{Z}_0^{\mathrm{c}}(h_1,\dots,h_k) = \sum_{x_1,\dots,x_k} \omega(x_1,\dots,x_k) \prod_{i=1}^{k} \left(\frac{1+x_i h_i}{2}\right) \,, \qquad [64]$$

$$\mathcal{Z}_0^{\mathrm{e}}(h,u) = \sum_x \left(\frac{1+xh}{2}\right)\left(\frac{1+xu}{2}\right) \,. \qquad [65]$$

Replica symmetric cavity equations: The simplest version of the cavity method implies the assumption of Replica Symmetry (RS). This is rigorously justified in our case thanks to the fact that we are considering a Bayes-Optimal model.

In such a case, the resulting self-consistent equations are relatively simpler:

$$\mathcal{P}^{RS}(h) = \sum_{d=0}^{\infty} p_d \int \left(\prod_{i=1}^{d} \mathrm{d}u_i \widehat{\mathcal{P}}^{RS}(u_i)\right) \delta(h - f(u_1,\dots,u_d)) \,, \qquad [66]$$

$$\widehat{\mathcal{P}}^{RS}(u) = \int \left(\prod_{i=1}^{k-1} \mathrm{d}h_i \mathcal{P}^{RS}(h_i)\right) \delta(u - g(h_1,\dots,h_{k-1})) \,.$$

[67]

where $\mathcal{P}^{RS}$ and $\widehat{\mathcal{P}}^{RS}$ are probability distributions defined on the messages h and u respectively.

Being self-consistency equations defined on probability distributions makes the computation of an analytical solution possible only in very restricted cases, while in practise one needs to use numerical techniques to find approximate solutions.

Still, *population dynamics* techniques (see for example (54)) have been shown to provide very good approximations when one is interested in observables defined as average quantities over $\mathcal{O}(N)$ cavity messages.

When the density of interactions α becomes large, the hypothesis underlying the Replica Symmetric assumption breaks down, and we have to consider the Replica Symmetry Breaking (RSB) phenomenon (55).

D.3. 1RSB and Tree reconstruction equations. The 1RSB cavity method aims to compute the potential

$$\Phi_1(m) = \lim_{N\to\infty} \frac{1}{N} \log\left(\sum_\gamma Z_\gamma^m\right) \qquad [68]$$

where m is the so-called Parisi parameter.

In their general formulation, the 1RSB equations are self-consistent equations defined on distributions over probability distributions. Focusing on the case $m=1$ allows simplifying considerably the equations, and the resulting formulas correspond to the so-called *Tree Reconstruction* equations (56). Moreover, the problem we are considering has a global spin-flip symmetry which allows us to simplify further the equations, which can be finally written as

$$\begin{aligned} Q_+^{(t+1)}(h) &= \sum_{d=0}^{\infty} p_d \int \left(\prod_{i=1}^{d} \mathrm{d}u_i \widehat{Q}_+^{(t)}(u_i)\right) \delta(h - f(u_1,\dots,u_d)) \ , \\ \widehat{Q}_+^{(t)}(u) &= \sum_{x_1,\dots,x_{k-1}} \tilde{p}(x_1,\dots,x_{k-1}|+) \int \left(\prod_{i=1}^{k-1} \mathrm{d}h_i Q_+^{(t)}(h_i)\right) \delta(u - g(x_1h_1,\dots,x_{k-1}h_{k-1})) \ , \end{aligned} \qquad [69]$$

where

$$\tilde{p}(x_1,\dots,x_{k-1}|+) = \frac{\omega(x_1,\dots,x_{k-1},+)}{\sum_{x'_1,\dots,x'_{k-1}} \omega(x'_1,\dots,x'_{k-1},+)} = \frac{\sum_{p=0}^{k-1} \omega_p \, \mathbb{I}\left[\sum_{i=1}^{k-1} x_i = k-1-2p\right]}{\sum_{p=0}^{k-1} \binom{k-1}{p} \omega_p} \ . \qquad [70]$$

Similar as before, we can compute the RS free entropy for the planted problem (53) as

$$\begin{aligned} \Phi_{\mathrm{RS}} = &\sum_{d=0}^{\infty} p_d \int \left(\prod_{i=1}^{d} \mathrm{d}u_i \widehat{Q}_+(u_i)\right) \ln \mathcal{Z}_0^{\mathrm{v}}(u_1,\dots,u_d) + \alpha \sum_{x_1,\dots,x_k} p(x_1,\dots,x_k) \int \left(\prod_{i=1}^{k} \mathrm{d}h_i Q_+(h_i)\right) \ln \mathcal{Z}_0^{\mathrm{c}}(x_1h_1,\dots,x_kh_k) \\ &- \alpha k \int \mathrm{d}h \, \mathrm{d}u Q_+(h) \widehat{Q}_+(u) \ln \mathcal{Z}_0^{\mathrm{e}}(h,u) \ , \end{aligned} \qquad [71]$$

where

$$p(x_1,\dots,x_k) = \frac{\omega(x_1,\dots,x_k)}{\sum_{x'_1,\dots,x'_k} \omega(x'_1,\dots,x'_k)} \ . \qquad [72]$$

and $\mathcal{Z}_0^{\mathrm{c}}$, $\mathcal{Z}_0^{\mathrm{e}}$ and $\mathcal{Z}_0^{\mathrm{v}}$ are the local partitions reported in Eq. [64], Eq. [65] and Eq. [63] respectively.

Finally, the order parameter for the problem, corresponding to the definition in Eq. [7], can be computed from the population dynamics simulations as

$$\chi = \int \mathrm{d}h Q_+(h) h^2 \, . \qquad [73]$$

D.4. Tilted and Pinned measures. In the preceding section, we presented the classical k-NAESAT model and how its properties can be studied using the cavity method. Now, let us consider the tilted and pinning measures and see how this changes the previous equations. As far as we know, the Cavity equations and phase diagrams shown in the following are new and were not presented in this format in any previous work.

Tilted measure: Starting with the **tilted measure**, the added tilting field results directly in the function defining the messages going from the factor nodes to the variable nodes, which is modified to

$$f^{\text{tilt}}(u_1,\ldots,u_d) = \frac{e^{2\gamma(\gamma+z)}\prod_{i=1}^{d}(1+u_i) - \prod_{i=1}^{d}(1-u_i)}{e^{2\gamma(\gamma+z)}\prod_{i=1}^{d}(1+u_i) + \prod_{i=1}^{d}(1-u_i)}\,, \quad z\sim\mathcal{N}(0,1)\,, \tag{74}$$

while the function g, defined in Eq. [61], is not modified. In terms of the partition function, the only local term that is modified is given by

$$\mathcal{Z}_0^{\text{v}}(u_1,\ldots,u_d) = e^{\gamma(\gamma+z)}\prod_{i=1}^{d}\left(\frac{1+u_i}{2}\right) + e^{-\gamma(\gamma+z)}\prod_{i=1}^{d}\left(\frac{1-u_i}{2}\right)\,, \quad z\sim\mathcal{N}(0,1)\,. \tag{75}$$

while the other two terms remain the same.

Pinning measure: For the **pinning measure**, again the function g remains the same, but now we have

$$f^{\text{pinn}}(u_1,\ldots,u_d) = \begin{cases} 1 & \text{with probability } \theta \\ \frac{\prod_{i=1}^{d}(1+u_i)-\prod_{i=1}^{d}(1-u_i)}{\prod_{i=1}^{d}(1+u_i)+\prod_{i=1}^{d}(1-u_i)} & \text{with probability } 1-\theta \end{cases}, \tag{76}$$

and again the only contribution to the partition function that is modified is

$$\mathcal{Z}_0^{\text{v}}(u_1,\ldots,u_d) = \begin{cases} \prod_{i=1}^{d}\left(\frac{1+u_i}{2}\right) & \text{with probability } \theta \\ \prod_{i=1}^{d}\left(\frac{1+u_i}{2}\right) + \prod_{i=1}^{d}\left(\frac{1-u_i}{2}\right) & \text{with probability } 1-\theta \end{cases}. \tag{77}$$

Phase diagrams: In Fig. S11 we present the phase diagrams for the k-NAESAT problem, considering the case $k = 5$. Compared

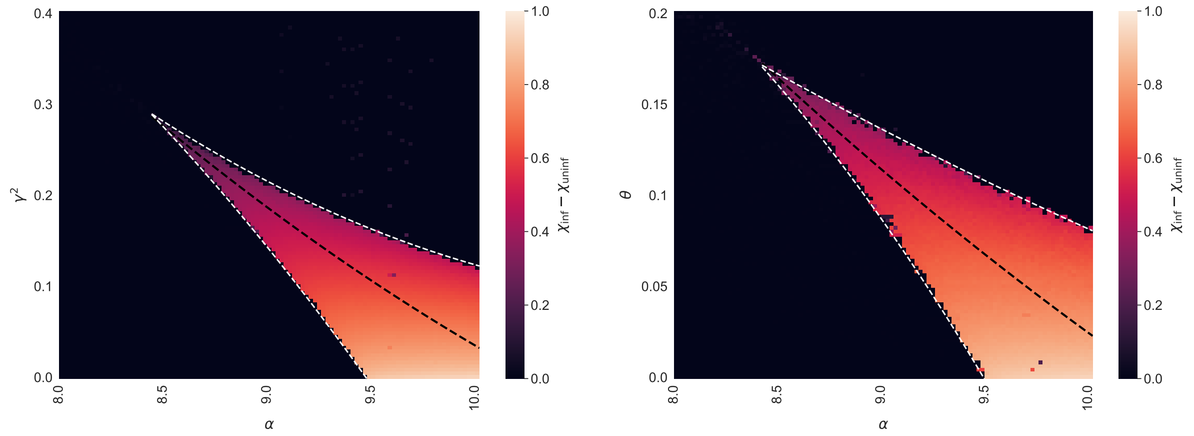

Fig. S11. Phase diagrams for flow-based sampling (left) and autoregressive-based sampling (right) for the k-NAESAT model with $k = 5$. On the x-axis we put the constraints-to-variables ratio $\alpha = M/N$ and on the y-axis the ratio $\gamma^2 = \alpha^2/\beta^2$ (left) and the decimated ratio θ (right). We compute the order parameter χ, defined in Eq. [73], both from an uninformed and an informed initialization, and we plot the difference between the two. The dashed white lines are the *spinodal lines*, while the dashed black one is the *IT threshold*, both defined at the beginning of the section. All dashed lines were computed using a polynomial fit. In both plots we have that the dynamical transition is at $\alpha_d \approx 9.465$, the Kauzmann (or condensation) transition is at $\alpha_K \approx 10.3$, while the tri-critical points are at $\alpha_{\text{tri}} \approx 8.4$ for both flow-based and autoregressive based sampling. Thus, within our numerical precision, the two methods seems to be equally efficient.

to the plots reported in the main text, here we display directly the difference $\chi_{\text{inf}} - \chi_{\text{uninf}}$, so that the coloured zones of the plots are the ones displaying multiple fixed points, as opposed to the black ones. We furthermore draw as white dashed lines the spinodal points, and as a black dashed line the IT threshold, both defined at the beginning of Appendix 3.

Compared to the previous models, defined on dense graphs, here we don't have some fixed point equations defined on an $O(1)$ number of scalar parameter, but instead some self-consistent equations on probability distributions. This clearly makes it harder to have a precise estimate of the tri-critical points, both for the tilted and the pinned measures. For the precision we were able to achieve, both for flow-based sampling and for autoregressive-based sampling the tri-critical point appears to be around $\alpha_{\text{tri}} \approx 8.4$, and we are not able to state if one of the two methods has a smaller gap compared to the other and thus if there is a range in α where it is able to sample efficiently where the other can not. A more careful analysis is needed to clarify this point.

4. Bayes optimal inference: Concentration, Replica Symmetry and Nishimori identities

We shall briefly recall here some of the important properties of the measure associated with optimal Bayesian denoising, that we are using in the main text. All of these properties are well known in the literature, but we recall them for completeness

The first one are the well-known Nishimori identities (8, 9, 57), which are valid for any Bayesian posterior estimation problem where one observes a variable Y sampled from $P(Y|X)$, and attempt to reconstruct X by computing the posterior average. We reproduce the theorem and the proof here

Theorem 1 (Nishimori Identity). *Let $X^{(1)}, \ldots, X^{(k)}$ be k i.i.d. samples (given Y) from the distribution $P(X = \cdot \mid Y)$. Denoting $\langle\cdot\rangle$ the "Boltzmann" expectation, that is the average with respect to the $P(X = \cdot \mid Y)$, and $\mathbb{E}[\cdot]$ the "Disorder" expectation, that is with respect to (X^*, Y). Then for all continuous bounded function f we can switch one of the copies for X^*:*

$$\mathbb{E}\left[\left\langle f\left(Y, X^{(1)}, \ldots, X^{(k-1)}, X^{(k)}\right)\right\rangle_k\right] = \mathbb{E}\left[\left\langle f\left(Y, X^{(1)}, \ldots, X^{(k-1)}, X^*\right)\right\rangle_{k-1}\right] \quad [78]$$

Proof. The proof is a consequence of Bayes theorem and of the fact that both x^* and any of the copy $X^{(k)}$ are distributed from the posterior distribution. Denoting more explicitly the Boltzmann average over k copies for any function g as

$$\left\langle g(X^{(1)}, \ldots, X^{(k)})\right\rangle_k =: \int \prod_{i=1}^{k} \mathrm{d}x_i P(x_i|Y) g(X^{(1)}, \ldots, X^{(k)}) \quad [79]$$

we have, starting from the right-hand side

$$\begin{aligned}
&\mathbb{E}_{Y,X^*}\left[\left\langle f\left(Y, X^{(1)}, \ldots, X^{(k-1)}, X^*\right)\right\rangle_{k-1}\right] \\
&= \int dx^* dy P(x^*|Y) P(Y) \left\langle f\left(Y, X^{(1)}, \ldots, X^{(k-1)}, X^*\right)\right\rangle_{k-1} \\
&= \mathbb{E}_Y \int \mathrm{d}x^k P(x^k|y) \left\langle f\left(Y, X^{(1)}, \ldots, X^{(k-1)}, X^k\right)\right\rangle_{k-1} \\
&= \mathbb{E}_Y \left[\left\langle f\left(Y, X^{(1)}, \ldots, X^{(k-1)}, X^{(k)}\right)\right\rangle_k\right]
\end{aligned}$$

□

In particular, we have the relation $\mu = \chi$, as stated in the main text.

We now move to the specific case of Gaussian denoising, and more specifically to the measure:

$$P_\gamma(\mathbf{x}) = \frac{1}{Z_n} \exp\left(\gamma^2 \langle \mathbf{x_0}, \mathbf{x}\rangle + \gamma\langle \mathbf{z}, \mathbf{x}\rangle - \frac{\gamma^2}{2}\|\mathbf{x}\|^2\right) P_0(\mathbf{x}) \quad [80]$$

The first identity is that the derivative of the free entropy associated with this problem is simply the expected overlap μ (This is often called in slightly different context the I-MMSE theorem (58)) and the second derivative the (Boltzmann) variance of the overlap (this is often called the Fluctuation-Dissipation theorem in statistical mechanics):

Lemma 1 (First(I-MMSE theorem(59)) and second (FDT theorem (60)) derivative of the free entropy). *Consider the free entropy density associated with the measure in Eq. [80]:*

$$f_N = \frac{1}{N}\mathbb{E}[\log Z_n(\gamma)] \quad [81]$$

then

$$\partial_\gamma f_n = \gamma\mu(\gamma) \equiv \frac{\gamma}{N}\mathbb{E}[\langle \mathbf{x}(\gamma)\rangle \mathbf{x}_0] \quad [82]$$

and

$$\partial_\gamma^2 f_N = N\gamma^2 \mathbb{E}\left[\left(\langle (\frac{\mathbf{x}(\gamma)\cdot \mathbf{x}_0}{N})^2\rangle\right) - \left(\frac{\langle \mathbf{x}(\gamma)\rangle \mathbf{x}_0}{N}\right)^2\right] = N\gamma^2\mathbb{E}[\mathrm{var}\left(\frac{\mathbf{x}\cdot\mathbf{x}_0}{N}\right)] \quad [83]$$

Proof. The proof is a direct application of Nishimori identities together with Stein lemma (which states that $\mathbb{E}_Z z g(Z) = \mathbb{E} g'(z)$ for a Gaussian random variable Z), which we reproduce here:

$$\begin{aligned}
\partial_\gamma f_n &= \mathbb{E}\int d\mathbf{x} P_\gamma(\mathbf{x}) \frac{2\gamma \mathbf{x}_0\cdot\mathbf{x} + \mathbf{x}\cdot\mathbf{z} - \gamma \mathbf{x}\cdot\mathbf{x}}{N\mathbf{z}(\gamma)} && [84]\\
&= 2\gamma\mu - \gamma\mathbb{E}\langle\frac{\mathbf{x}\cdot\mathbf{x}}{N}\rangle - \mathbb{E}\int d\mathbf{x} P_\gamma(X)\frac{X\cdot Z}{N\mathbf{z}(\gamma)} && [85]\\
&= 2\gamma\mu - \gamma\mathbb{E}\langle\frac{\mathbf{x}\cdot\mathbf{x}}{N}\rangle + \gamma\mathbb{E}\langle\frac{\mathbf{x}\cdot\mathbf{x}}{N}\rangle - \gamma\frac{1}{N}\langle\mathbf{x}\rangle\cdot\langle\mathbf{x}\rangle && [86]\\
&= 2\gamma\mu - \chi = \gamma\mu && [87]
\end{aligned}$$

where we use Stein lemma in Eq. [86] and Nishimori Eq. [87]. The second derivative identity is obtained along the same line by deriving twice with respect to γ. □

This lemma in turn implies that the concentration of the overlap μ, a trick often used in the literature of mathematical physics when proving the replica equation (see e.g. (20)):

Theorem 2 (Concentration of overlaps)**.** *Almost everywhere in gamma, we have for some $K(\gamma)$, that*

$$\mathbb{E}[\mathrm{var}\left(\frac{\mathbf{x}\cdot\mathbf{x}_0}{N}\right)] \to_{N\to\infty} 0$$

Proof. From the derivative, we have that

$$\int_{\gamma_1}^{\gamma_2} \mathrm{var}_\gamma\left(\frac{\mathbf{x}\cdot\mathbf{x}_0}{N}\right) = \frac{1}{\gamma^2 N}(\mu(\gamma_2)-\mu(\gamma_1)) \leq \frac{K}{N} \qquad [88]$$

where we have assumed that μ is bounded by some constant (which is the case for the discrete variables discussed in this paper where $-1<\mu<1$). As a consequence, almost everywhere in γ, the variance of the overlap must vanish. □

Additionally, one can also prove the concentration of the variance with respect to the disorder, see (20).
Finally, similar results exist in the case of "pinning" (21, 22).

5. Analysis of Algorithm 1

In this section, we provide a theoretical analysis of the performance of Algorithm 1 for the "efficient" regime in Figure 1. In these regimes, we conjecture being able to approximate perfect denoising during the interpolation path. Therefore, to simplify our analysis, we assume access to a perfect denoiser and primarily focus on the validity of the continuous-time limit and the effect of discretization. Additional approximation and discretization errors due to the denoiser, such as AMP can be incorporated in our analysis through a straightforward manner. For a rigorous analysis of the approximation error and Lipschitzness of the AMP iterates in the case of the SK and spiked matrix models, we refer the reader to (6, 61).

Lastly, throughout the analysis, we shall assume that Algorithm 1 is run from time $t=1$ to $t=\epsilon$ for some $\epsilon>0$. This ensures that we can avoid singularities at $t=0$, while choosing ϵ arbitrarily close to 0 allows us to approximate the target measure up to arbitrary accuracy.

Recall the definition of the "tilted" posterior measure at time t:

$$P(\mathbf{x}|\mathbf{y}(t)=\mathbf{Y}_t) = \frac{1}{Z(\mathbf{Y}_t)}\exp\left(\frac{\alpha(t)}{\beta(t)^2}\langle\mathbf{Y}_t,\mathbf{x}\rangle - \frac{\alpha(t)^2}{2\beta(t)^2}||\mathbf{x}||^2\right)P_0(\mathbf{x}). \qquad [89]$$

Here the factor $\frac{1}{Z(\mathbf{Y}_t)}\exp\left(\frac{\alpha(t)}{\beta(t)^2}\langle\mathbf{Y}_t,\mathbf{x}\rangle - \frac{\alpha(t)^2}{2\beta(t)^2}||\mathbf{x}||^2\right)$ is interpreted as the Radon-Nikodym derivative of $P(\mathbf{x}|\mathbf{y}(t)$ w.r.t P_0. Throughout the present section, we shall denote $P(\mathbf{x}|\mathbf{y}(t)=\mathbf{Y}_t)$ by $P_{t,\mathbf{Y}_t}$. We shall assume that $\alpha(t),\beta(t)$ are continuously-differentiable.

A. Fluctuation-dissipation and Lipschitzness. A crucial quantity related to the well-posedness of the continuity equation (Eq. [4]) and the validity of Algorithm 1 is the Lipschitz constant of the vector field. We therefore start by present a preliminary result, which can be interpreted as an instance of the Fluctuation-dissipation theorem:

Lemma 2. *Suppose that the measure P_0 has compact support. Then the Jacobian of the vector $\frac{\partial b(\mathbf{y},t)}{\partial \mathbf{y}}$ is related to the covariance of the tilted measure $P_{t,\mathbf{Y}_t}=P(\mathbf{x}|\mathbf{y}(t)=\mathbf{Y}_t)$ as follows:*

$$\frac{\partial b(\mathbf{y},t)}{\partial\mathbf{y}}|_{\mathbf{y}=\mathbf{Y_t}} = \frac{\alpha(t)}{\beta(t)^2}(\dot\alpha(t)-\frac{\dot\beta(t)\alpha(t)}{\beta(t)})\,\mathrm{Covar}[P_{t,\mathbf{Y}_t}] + \frac{\dot\beta(t)}{\beta(t)} \qquad [90]$$

Proof. We have, from Eq. [5]:

$$\begin{aligned} b(\mathbf{y},t) &= \mathbb{E}[\dot\alpha(t)\mathbf{x_0}+\dot\beta(t)z|\mathbf{y}(t)=\mathbf{y}] \\ &= \mathbb{E}[\dot\alpha(t)\mathbf{x_0}+\frac{\dot\beta(t)}{\beta(t)}(\mathbf{y}(t)-\alpha(t)\mathbf{x}_0)|\mathbf{y}(t)=\mathbf{y}] \\ &= (\dot\alpha(t)-\frac{\dot\beta(t)\alpha(t)}{\beta(t)})\mathbb{E}[\mathbf{x_0}|\mathbf{y}(t)=\mathbf{y}]+\frac{\dot\beta(t)}{\beta(t)}\mathbf{y}. \end{aligned} \qquad [91]$$

where we used the relation in Eq. [1]. Next, we have through the expression for the posterior measure $P_{t,\mathbf{Y}_t}$:

$$\mathbb{E}[\mathbf{x_0}|\mathbf{y}(t)=\mathbf{y}] = \mathbb{E}_{P_0}\left[\frac{1}{Z(\mathbf{y})}\mathbf{x}\exp\left(\frac{\alpha(t)}{\beta(t)^2}\langle\mathbf{y},\mathbf{x}\rangle - \frac{\alpha(t)^2}{2\beta(t)^2}||\mathbf{x}||^2\right)\right]. \qquad [92]$$

Using the boundedness of the support of P_0 and dominated convergence theorem, we may differentiate the L.H.S inside the expectation. We obtain that for all $t\in(0,1]$, $\mathbb{E}[\mathbf{x_0}|\mathbf{y}(t)=\mathbf{Y}_t]$ is differentiable w.r.t $\mathbf{Y}_t$ with the Jacobian given by $\frac{\alpha(t)}{\beta(t)^2}\mathrm{Covar}[P_{t,\mathbf{Y}_t}]$. Substituting into Eq. [91] completes the proof.

□

B. Derivation of the ODE (Eq. [6]). We start by presenting an informal derivation of the continuity equation, borrowed from (62). Recall that, by definition, $\mathbf{y}(t) = \alpha(t)\mathbf{x}_0 + \beta(t)\mathbf{z}$. The density $\rho(\mathbf{y}, t)$ can be expressed as:

$$\rho(\mathbf{y}, t) = \int_{\mathbb{R}^N \times \mathbb{R}^N} \delta(\mathbf{y} - \mathbf{y}(\mathbf{t}))\rho_{\mathbf{0}}(\mathbf{x_0})\rho_\gamma(\mathbf{z})\mathbf{dx_0dz}, \quad [93]$$

where ρ_γ denotes the density of the standard Gaussian measure $\gamma = \mathcal{N}(\mathbf{0}, \mathbb{I}_N)$. Differentiating both sides of Eq. [93] yields:

$$\begin{aligned}\partial_t\rho(\mathbf{y}, t) &= -\int_{\mathbb{R}^N \times \mathbb{R}^N} \nabla\delta(\mathbf{y} - \mathbf{y}(\mathbf{t})) \cdot \partial_\mathbf{t}\mathbf{y}(\mathbf{t})\rho_{\mathbf{0}}(\mathbf{x_0})\rho_\gamma(\mathbf{z})\mathbf{dx_0dz} \\ &= -\nabla \cdot \int_{\mathbb{R}^N \times \mathbb{R}^N} \delta(\mathbf{y} - \mathbf{y}(\mathbf{t}))\partial_\mathbf{t}\mathbf{y}(\mathbf{t})\rho_{\mathbf{0}}(\mathbf{x_0})\rho_\gamma(\mathbf{z})\mathbf{dx_0dz} \\ &= -\nabla \cdot \left(\int_{\mathbb{R}^N \times \mathbb{R}^N} \left(\delta(\mathbf{y} - \mathbf{y}(t))\partial_t\mathbf{y}(t)\frac{\rho_0(\mathbf{x_0})\rho_\gamma(\mathbf{z})}{\rho(\mathbf{y}, t)}d\mathbf{x_0}d\mathbf{z}\right)\rho(\mathbf{y}, t)\right)\end{aligned} \quad [94]$$

Eq. [4] is then obtained by noticing that $\int_{\mathbb{R}^d \times \mathbb{R}^d} \left(\delta(\mathbf{y} - \mathbf{y}(\mathbf{t}))\partial_\mathbf{t}\mathbf{y}(\mathbf{t})\frac{\rho_\mathbf{0}(\mathbf{x_0})\rho_\gamma(\mathbf{z})}{\rho(\mathbf{y},\mathbf{t})}\mathbf{dx_0dz}\right)$ equals $b(\mathbf{y}, t)$ defined by Eq. [5].

We refer to (62, 63) for the complete derivation based on the above approach.

We next prove that the pushforward measure obtained after applying the flow defined by the ODE in Eq. [6] to initial Gaussian noise $\mathbf{z}$ results in a sample from the target measure P_0. For the sake of completeness, we include an alternative derivation for Eq. [4] in our proof, based on the weak form of the continuity equation. Our result allows P_0 to be a discrete measure, by restricting the time to $t \in (0, 1)$.

Lemma 3. *Let ϵ be an arbitrarily-fixed real in $(0, 1)$. Let $\mathbf{Y_t}(\mathbf{z})$ denote the flow associated with the ODE in Eq. [6] starting from $t = 1$ to $t = \epsilon$ with $\mathbf{z} \sim \gamma$, where γ denotes the standard Gaussian measure $\gamma = \mathcal{N}(\mathbf{0}, \mathbb{I}_N)$. Suppose that P_0 has bounded support. Then, the pushforward measure $\mathbf{Y_t}_\#\gamma$ at any time $t \in (0, 1]$ equals the measure corresponding to the law P_t of the interpolant $\mathbf{y}(t)$ defined by Eq. [1].*

Proof. Let $\psi \in C_c^\infty(\mathbb{R}^d)$ be an arbitrary test function. Using the change of variables formula, the expectation of ψ w.r.t the measure μ_t at time t can be expressed as:

$$\int_{\mathbb{R}^N} \psi(\mathbf{y})dP_t(\mathbf{y}) = \int_{\mathbb{R}^N \times \mathbb{R}^N} \psi(\mathbf{y}(t))dP_0(\mathbf{x}_0)d\gamma_z(\mathbf{z}), \quad [95]$$

where $\mathbf{y}(t)$ is defined as a measurable function of $\mathbf{x}_0, \mathbf{z}$ through Eq. [1].

Therefore, P_t evolves in a distributional sense as follows:

$$\int_{\mathbb{R}^N} \psi(\mathbf{y})\partial_t dP_t(\mathbf{y}) = \frac{d\int_{\mathbb{R}^N} \psi(\mathbf{y})dP_t(\mathbf{y})}{dt} = \int_{\mathbb{R}^N \times \mathbb{R}^N} \nabla\psi(\mathbf{y}(t)) \cdot \partial_t\mathbf{y}(t)dP_0(\mathbf{x}_0)d\gamma_z(\mathbf{z}). \quad [96]$$

Recall that:

$$b(\mathbf{y}, t) = \mathbb{E}[\partial_t\mathbf{y}(t)|\mathbf{y}(t) = \mathbf{y}]. \quad [97]$$

Using the definition of the conditional expectation, and the change of variables formula, we have:

$$\begin{aligned}\int_{\mathbb{R}^N \times \mathbb{R}^N} \nabla\psi(\mathbf{y}(t)) \cdot \partial_t\mathbf{y}(t)dP_0(\mathbf{x}_0)d\gamma_z(\mathbf{z}) &= \int_{\mathbb{R}^N} \nabla\psi(\mathbf{y}).\mathbb{E}[\partial_t\mathbf{y}(t)|\mathbf{y}(t) = \mathbf{y}]dP_t(\mathbf{y}) \\ &= \int_{\mathbb{R}^N} \nabla\psi(\mathbf{y}) \cdot b(\mathbf{y}, t)dP_t(\mathbf{y}) \\ &= -\int_{\mathbb{R}^N} \psi(\mathbf{y})\nabla.(b(\mathbf{y}, t)P_t(\mathbf{y}))d\mathbf{y},\end{aligned}$$

where we used the compactness of the support of $\psi(x)$ and the distributional definition of the divergence operator ∇. Substituting in Eq. [96], we obtain:

$$\int_{\mathbb{R}^N} \psi(\mathbf{y})\partial_t dP_t(\mathbf{y}) = -\int_{\mathbb{R}^N} \psi(\mathbf{y})\nabla.(b(\mathbf{y}, t)P_t(\mathbf{y}))d\mathbf{y}. \quad [98]$$

Furthermore, for all $t > \epsilon$ for fixed $\epsilon > 0$, we have $\beta(t) > 0$. Then, Lemma 2 implies that $b(\mathbf{y}, t)$ is Lipschitz w.r.t $\mathbf{y}$. Thus, the probability flow $\mathbf{Y_t}$ for $t \in [\epsilon, 1]$ exists and is unique. Since both the push-forward measure $\mathbf{Y_t}\#\gamma$ and the law of $\mathbf{y}(t)$ i.e. P_t satisfy the continuity equation with velocity field $b(\mathbf{y}, t)$ (Lemma 4.1.1. in (64)). By the uniqueness of the solution of the continuity equation (see e.g. (65)), $b(t, x)_\#\mu(z) = \mu_t$. ☐

C. Sampling Guarantees. In this section, we quantify the effect of discretization errors in the velocity field, leading to sampling guarantees for Algorithm 1. Again, we fix a parameter ϵ lying in $(0, 1)$ We rely on the following assumptions:

Assumption 1. *The measure P_0 has compact support lying in a sphere with radius bounded as $\mathcal{O}(\sqrt{N})$*

$$\mathrm{Supp}(P_0) \subseteq \mathbb{S}^{N-1}(\sqrt{N}R), \qquad [99]$$

for some R independent of N.

Assumption 2. *The spectral norm of the vector field's Jacobian $\frac{\partial b(\mathbf{Y}_t,t)}{\partial \mathbf{Y}_t}$ w.r.t $\mathbf{Y}$ is uniformly bounded, i.e. $\|\frac{\partial b(\mathbf{Y},t)}{\partial \mathbf{Y}}\|_2 \leq L, \forall t \in (\epsilon, 1), \forall \mathbf{Y} \in \mathbb{R}^d$ for some $L \geq 0$.*

Assumption 3. *$\|\frac{\partial b(\mathbf{Y},t)}{\partial t}\|$ is uniformly bounded by $M\sqrt{N}$ in $\mathbf{Y}, t$ for $t \in (\epsilon, 1)$, for some $M \geq 0$.*

We have the following error bounds for the discretization error associated to the flow:

Lemma 4. *Consider the ODE defined by Eq. [6] i.e $\frac{d\mathbf{Y}}{dt} = b(\mathbf{Y}, t)$, with $\mathbf{Y} \in \mathbb{R}^N$. Let $\mathbf{Y}_t(\mathbf{z})$ denote the flow associated to the above ODE at time $t \in (0, 1]$ starting from some fixed $\mathbf{z} \in \mathbb{R}^N$ at time $t = 1$. Let $\mathbf{Y}_{\delta,t}(\mathbf{z})$ denote the iterates of the forward Euler method applied to the above ODE (in reverse time) starting from the same initialization $\mathbf{z}$ with step-size δ i.e, for $i \in \mathbb{N}$:*

$$\mathbf{Y}_{\delta,\delta(i-1)}(\mathbf{z}) = \mathbf{Y}_{\delta,\delta i}(\mathbf{z}) - \delta b(\mathbf{Y}_{\delta,\delta i}(\mathbf{z}), \delta i), \qquad [100]$$

with $\mathbf{z}$ fixed. Under Assumptions 1,2,3, there exists a constant $A(\epsilon)$ such that, for all $k \in \mathbb{N}, \mathbf{z} \in \mathbb{R}^N$ with $k\delta \geq \epsilon$:

$$\|\mathbf{Y}_{\delta,k\delta}(\mathbf{z}) - \mathbf{Y}_{k\delta}(\mathbf{z})\|_2 \leq (\frac{M + AL}{L})\sqrt{N}e^{Lk\delta}\delta, \qquad [101]$$

for small enough δ.

Proof. We first note that Assumption 1 and Lemma 2 imply that $b(\mathbf{Y}, t)$ is uniformly bounded for any fixed $\mathbf{z}$.Then, the above bound follows from standard analysis of the forward Euler method. See for example Chapter 7 in (66). □

Remark: The Lipschitzness of the posterior mean (optimal denoiser) w.r.t $\mathbf{Y}_t$ is expected to hold for the setups considered in our work in light of similar results proven in (6, 67). Furthermore, as Lemma 2 shows, it is equivalent to the boundedness of the covariance of the tilted measure. Similarly, Lemma 1 provides control over $\frac{\partial b(\mathbf{Y},t)}{\partial t}$ through the variance of the overlap.

We now prove that the proposed algorithm with sufficiently small step for the discretized ODE produces a distribution close to the target distribution in Wasserstein distance. The obtained bounds on the error between the ODE and the algorithm iterates can be related to the Wasserstein distance between the corresponding pushforward measures.

Theorem 3. *Let $P_{\mathrm{alg},\mathrm{N_{steps}}}$ denote the measure of the output produced by Algorithm 1 after $\mathrm{N_{steps}}$. Suppose that the vector field, for any $\eta > 0$, there exist $\mathrm{N_{steps}}$, independent of N, such that the normalized Wasserstein distance $\frac{1}{\sqrt{N}}W_2(P_{\mathrm{alg},\mathrm{N_{steps}}}, P_0) < \eta$.*

Proof. Let $0 < \epsilon < h$ be arbitrary. By definition, $\mathbf{Y}_{h,\epsilon}$ has law $P_{\mathrm{alg},\mathrm{N_{steps}}}$ while, from Lemma 3, $\mathbf{Y}_\epsilon$ equals in law $\mathbf{y}(\epsilon)$. Setting the same initialization $\mathbf{z}$ induces a coupling between the two measures. Recall that the 2-Wasserstein distance (64, 68) between two measures μ, ν on $\mathbb{R}^N$ is defined as $W_2^2(\mu, \nu) = \inf_{\Gamma(\mu,\nu)} \mathbb{E}\|\mathbf{X} - \mathbf{Y}\|^2$, where $\Gamma(\mu, \nu)$ denotes the set of couplings between μ, ν with $\mathbf{X}, \mathbf{Y}$ being distributed as μ, ν respectively. Therefore, we obtain:

$$W_2^2(P_{\mathrm{alg},\mathrm{N_{steps}}}, P_\epsilon) \leq \mathbb{E}\|\mathbf{Y}_{\delta,\epsilon}(\mathbf{z}) - \mathbf{Y}_\epsilon(\mathbf{z})\|^2. \qquad [102]$$

Lemma 4 then implies that by choosing a small enough step-size δ, or equivalently, with a large enough $\mathrm{N_{steps}}$, $W_2(P_{\mathrm{alg},\mathrm{N_{steps}}}, P_\epsilon)$ can be made arbitrarily small for any fixed epsilon.

We further have that $W_2(P_0, P_\epsilon) \to 0$ as $\epsilon \to 0$. By triangle inequality for W_2 (64, 68), we obtain:

$$W_2(P_0, P_\epsilon) \leq W_2(P_{\mathrm{alg},\mathrm{N_{steps}}}, P_\epsilon) + W_2(P_0, P_\epsilon). \qquad [103]$$

Now, first pick $\epsilon > 0$ such that $\frac{1}{\sqrt{N}}W_2(P_0, P_\epsilon) \leq \eta/2$. Subsequently, we pick $\mathrm{N_{steps}}$ such that $\frac{1}{\sqrt{N}}W_2(P_{\mathrm{alg},\mathrm{N_{steps}}}, P_\epsilon) \leq \eta/2$ to complete the proof. □

The above result establishes that Algorithm 1 samples from a distribution approximating the target distribution up to any desired accuracy, with a finite number of forward Euler steps ($\mathrm{N_{steps}}$), independent of the dimension N.

Remark: In the presence of a first-order phase transition during the interpolation path $\|\frac{\partial b(\mathbf{Y},t)}{\partial t}\|$ may grow as $\omega(\sqrt{N})$. This is apparent from Lemma 1 which relates $\frac{\partial b(\mathbf{Y},t)}{\partial t}$ to the variance of the overlap. This corresponds to M in Lemma 4 growing with N. Lemma 4 reveals that as long as this growth is polynomial in N, the discretization error can still be controlled by choosing h to be $1/\mathrm{poly}(N)$. This would lead only to an additional polynomial time factor in the time complexity of the algorithm. Therefore, with an oracle access to the denoiser, Algorithm 1 might be able to efficiently sample even in the "inefficient" phase. We believe this to be an interesting question for future research.

D. Sampling in the presence of phase transition with Optimal Denoiser. In the presence of a first-order phase transition, Assumptions 2 and 3 no longer hold. However, $\|\frac{\partial b(\mathbf{Y},t)}{\partial \mathbf{Y}}\|_2$ can be easily shown to be bounded by polynomial in N. Let $J = \frac{\partial b(\mathbf{Y},t)}{\partial \mathbf{Y}}$. Lemma 2 and Assumption 1 imply that almost surely over $\mathbf{x}$ with law $P_{t,\vec{Y}_t}$ satisfies, almost surely $\|\mathbf{x}\mathbf{x}^\top\| \leq N$. Using linearity of expectation and Jensen's inequality, we obtain:

$$\|\mathrm{Covar}[P_{t,\mathbf{Y}_t}]\| \leq \|\mathbb{E}_{P_{t,\mathbf{Y}_t}}\left[\mathbf{x}\mathbf{x}^\top\right]\| \leq \mathbb{E}_{P_{t,\mathbf{Y}_t}}\left[\|\mathbf{x}\mathbf{x}^\top\|\right] \leq N. \quad [104]$$

Therefore, Lemma 2 implies that $\|\frac{\partial b(\mathbf{Y},t)}{\partial \mathbf{Y}}\|_2$ for $t \in [1, \epsilon]$ is uniformly bounded by CN for some constant C. By Lemma 4, we obtain that using a step size $\delta = \mathcal{O}(1/N)$ suffices to sample from P upto any accuracy. This leads to an over-head in the time-complexity of a factor $\mathcal{O}(N)$, showing that the barrier towards efficient sampling results from the hardness of approximating the optimal denoiser, not from the discretization error itself. If one could approximate the optimal denoiser, sampling becomes efficient even in the "hard" phase.

References

1. T Lesieur, F Krzakala, L Zdeborová, Phase transitions in sparse pca in *2015 IEEE International Symposium on Information Theory (ISIT).* (IEEE), pp. 1635–1639 (2015).
2. J Ding, A Sly, N Sun, Satisfiability threshold for random regular nae-sat in *Proceedings of the forty-sixth annual ACM symposium on Theory of computing.* pp. 814–822 (2014).
3. A Perry, AS Wein, AS Bandeira, Statistical limits of spiked tensor models (2017).
4. T Lesieur, L Miolane, M Lelarge, F Krzakala, L Zdeborová, Statistical and computational phase transitions in spiked tensor estimation in *2017 IEEE International Symposium on Information Theory (ISIT).* pp. 511–515 (2017).
5. A Jaganath, P Lopatto, L Miolane, Statistical thresholds for tensor pca. *The Annals Appl. Probab.* **30**, 1910–1933 (2020).
6. A El Alaoui, A Montanari, M Sellke, Sampling from the sherrington-kirkpatrick gibbs measure via algorithmic stochastic localization in *2022 IEEE 63rd Annual Symposium on Foundations of Computer Science (FOCS).* (IEEE), pp. 323–334 (2022).
7. E Richard, A Montanari, A statistical model for tensor pca in *Advances in Neural Information Processing Systems*, eds. Z Ghahramani, M Welling, C Cortes, N Lawrence, K Weinberger. (Curran Associates, Inc.), Vol. 27, (2014).
8. H Nishimori, *Statistical Physics of Spin Glasses and Information Processing: An Introduction.* (Oxford University Press), (2001).
9. Y Iba, The nishimori line and bayesian statistics. *J. Phys. A: Math. Gen.* **32**, 3875 (1999).
10. A Montanari, G Semerjian, Rigorous inequalities between length and time scales in glassy systems. *J. statistical physics* **125**, 23–54 (2006).
11. F Krzakala, L Zdeborová, Hiding quiet solutions in random constraint satisfaction problems. *Phys. review letters* **102**, 238701 (2009).
12. L Zdeborová, F Krzakala, Generalization of the cavity method for adiabatic evolution of gibbs states. *Phys. Rev. B* **81**, 224205 (2010).
13. KM Wong, H Nishimori, Error-correcting codes and image restoration with multiple stages of dynamics. *Phys. Rev. E* **62**, 179 (2000).
14. AE Alaoui, A Montanari, M Sellke, Shattering in pure spherical spin glasses (2023).
15. F Guerra, Interpolation and comparison methods in the mean field spin glass model in *Trends in Contemporary Mathematics*, eds. V Ancona, E Strickland. (Springer International Publishing, Cham), pp. 1–12 (2014).
16. D Panchenko, The parisi ultrametricity conjecture (2015).
17. SB Korada, N Macris, Exact solution of the gauge symmetric p-spin glass model on a complete graph. *J. Stat. Phys.* **136**, 205–230 (2009).
18. J Barbier, N Macris, The adaptive interpolation method: a simple scheme to prove replica formulas in bayesian inference. *Probab. theory related fields* **174**, 1133–1185 (2019).
19. J Barbier, F Krzakala, N Macris, L Miolane, L Zdeborová, Optimal errors and phase transitions in high-dimensional generalized linear models. *Proc. Natl. Acad. Sci.* **116**, 5451–5460 (2019).
20. J Barbier, Overlap matrix concentration in optimal bayesian inference. *Inf. Inference: A J. IMA* **10**, 597–623 (2021).
21. E Abbe, A Montanari, Conditional random fields, planted constraint satisfaction and entropy concentration in *International Workshop on Approximation Algorithms for Combinatorial Optimization.* (Springer), pp. 332–346 (2013).
22. A Coja-Oghlan, F Krzakala, W Perkins, L Zdeborová, Information-theoretic thresholds from the cavity method in *Proceedings of the 49th Annual ACM SIGACT Symposium on Theory of Computing, STOC 2017, Montreal, QC, Canada, June 19-23, 2017*, eds. H Hatami, P McKenzie, V King. (ACM), pp. 146–157 (2017).
23. T Lesieur, F Krzakala, L Zdeborová, Constrained low-rank matrix estimation: Phase transitions, approximate message passing and applications. *J. Stat. Mech. Theory Exp.* **2017**, 073403 (2017).
24. SS Mannelli, et al., Marvels and pitfalls of the langevin algorithm in noisy high-dimensional inference. *Phys. Rev. X* **10**, 011057 (2020).
25. Y Deshpande, A Montanari, Information-theoretically optimal sparse pca in *2014 IEEE International Symposium on Information Theory.* (IEEE), pp. 2197–2201 (2014).
26. M Dia, et al., Mutual information for symmetric rank-one matrix estimation: A proof of the replica formula. *Adv. Neural Inf. Process. Syst.* **29** (2016).

27. M Lelarge, L Miolane, Fundamental limits of symmetric low-rank matrix estimation. *Probab. Theory Relat. Fields* **173**, 859–929 (2019).
28. DL Donoho, A Maleki, A Montanari, Message-passing algorithms for compressed sensing. *Proc. Natl. Acad. Sci.* **106**, 18914–18919 (2009).
29. M Mézard, G Parisi, MA Virasoro, *Spin glass theory and beyond: An Introduction to the Replica Method and Its Applications.* (World Scientific Publishing Company) Vol. 9, (1987).
30. M Gabrié, V Dani, G Semerjian, L Zdeborová, Phase transitions in the q-coloring of random hypergraphs. *J. Phys. A: Math. Theor.* **50**, 505002 (2017).
31. CM Stein, Estimation of the Mean of a Multivariate Normal Distribution. *The Annals Stat.* **9**, 1135 – 1151 (1981).
32. DL Donoho, M Gavish, IM Johnstone, Optimal shrinkage of eigenvalues in the spiked covariance model. *Annals statistics* **46**, 1742 (2018).
33. J Baik, JW Silverstein, Eigenvalues of large sample covariance matrices of spiked population models. *J. multivariate analysis* **97**, 1382–1408 (2006).
34. S Rangan, AK Fletcher, Iterative estimation of constrained rank-one matrices in noise in *2012 IEEE International Symposium on Information Theory Proceedings.* (IEEE), pp. 1246–1250 (2012).
35. F Krzakala, J Xu, L Zdeborová, Mutual information in rank-one matrix estimation in *2016 IEEE Information Theory Workshop (ITW).* (IEEE), pp. 71–75 (2016).
36. T Lesieur, F Krzakala, L Zdeborová, Mmse of probabilistic low-rank matrix estimation: Universality with respect to the output channel in *2015 53rd Annual Allerton Conference on Communication, Control, and Computing (Allerton).* (IEEE), pp. 680–687 (2015).
37. A El Alaoui, F Krzakala, Estimation in the spiked wigner model: a short proof of the replica formula in *2018 IEEE International Symposium on Information Theory (ISIT).* (IEEE), pp. 1874–1878 (2018).
38. DJ Thouless, PW Anderson, RG Palmer, Solution of'solvable model of a spin glass'. *Philos. Mag.* **35**, 593–601 (1977).
39. E Bolthausen, An iterative construction of solutions of the tap equations for the sherrington–kirkpatrick model. *Commun. Math. Phys.* **325**, 333–366 (2014).
40. J Baik, G Ben Arous, S Péché, Phase transition of the largest eigenvalue for nonnull complex sample covariance matrices. *The Annals Probab.* **33**, 1643–1697 (2005).
41. B Derrida, Random-energy model: Limit of a family of disordered models. *Phys. Rev. Lett.* **45**, 79 (1980).
42. DJ Gross, M Mézard, The simplest spin glass. *Nucl. Phys. B* **240**, 431–452 (1984).
43. TR Kirkpatrick, D Thirumalai, Dynamics of the structural glass transition and the p-spin—interaction spin-glass model. *Phys. review letters* **58**, 2091 (1987).
44. T Kirkpatrick, P Wolynes, Connections between some kinetic and equilibrium theories of the glass transition. *Phys. Rev. A* **35**, 3072 (1987).
45. G Biroli, JP Bouchaud, A Cavagna, TS Grigera, P Verrocchio, Thermodynamic signature of growing amorphous order in glass-forming liquids. *Nat. Phys.* **4**, 771–775 (2008).
46. G Biroli, JP Bouchaud, *The Random First-Order Transition Theory of Glasses: A Critical Assessment.* (John Wiley & Sons, Ltd), pp. 31–113 (2012).
47. P Charbonneau, J Kurchan, G Parisi, P Urbani, F Zamponi, Glass and jamming transitions: From exact results to finite-dimensional descriptions. *Annu. Rev. Condens. Matter Phys.* **8**, 265–288 (2017).
48. A Crisanti, HJ Sommers, The spherical p-spin interaction spin glass model: the statics. *Zeitschrift für Physik B Condens. Matter* **87**, 341–354 (1992).
49. J Ding, A Sly, N Sun, Satisfiability threshold for random regular nae-sat. *Commun. Math. Phys.* **341**, 435–489 (2016).
50. A Coja-Oghlan, L Zdeborová, The condensation transition in random hypergraph 2-coloring in *Proceedings of the Twenty-Third Annual ACM-SIAM Symposium on Discrete Algorithms, SODA 2012, Kyoto, Japan, January 17-19, 2012*, ed. Y Rabani. (SIAM), pp. 241–250 (2012).
51. T Castellani, V Napolano, F Ricci-Tersenghi, R Zecchina, Bicolouring random hypergraphs. *J. Phys. A: Math. Gen.* **36**, 11037 (2003).
52. F Ricci-Tersenghi, G Semerjian, L Zdeborová, Typology of phase transitions in bayesian inference problems. *Phys. Rev. E* **99**, 042109 (2019).
53. L Budzynski, F Ricci-Tersenghi, G Semerjian, Biased landscapes for random constraint satisfaction problems. *J. Stat. Mech. Theory Exp.* **2019**, 023302 (2019).
54. M Mezard, A Montanari, *Information, physics, and computation.* (Oxford University Press), (2009).
55. G Parisi, Infinite number of order parameters for spin-glasses. *Phys. Rev. Lett.* **43**, 1754–1756 (1979).
56. M Mézard, A Montanari, Reconstruction on trees and spin glass transition. *J. statistical physics* **124**, 1317–1350 (2006).
57. L Zdeborová, F Krzakala, Statistical physics of inference: Thresholds and algorithms. *Adv. Phys.* **65**, 453–552 (2016).
58. D Guo, S Shamai, S Verdú, , et al., The interplay between information and estimation measures. *Foundations Trends Signal Process.* **6**, 243–429 (2013).
59. D Guo, S Shamai, S Verdú, Mutual information and minimum mean-square error in gaussian channels. *IEEE transactions on information theory* **51**, 1261–1282 (2005).
60. R Kubo, The fluctuation-dissipation theorem. *Reports on progress physics* **29**, 255 (1966).
61. A Montanari, Y Wu, Posterior sampling from the spiked models via diffusion processes (2023).

62. MS Albergo, E Vanden-Eijnden, Building normalizing flows with stochastic interpolants in *The Eleventh International Conference on Learning Representations, ICLR 2023, Kigali, Rwanda, May 1-5, 2023.* (OpenReview.net), (2023).
63. MS Albergo, NM Boffi, E Vanden-Eijnden, Stochastic interpolants: A unifying framework for flows and diffusions (2023).
64. A Figalli, F Glaudo, *An Invitation to Optimal Transport, Wasserstein Distances, and Gradient Flows.* (ETH Zurich), (2021).
65. L Ambrosio, *Transport Equation and Cauchy Problem for Non-Smooth Vector Fields.* (Springer Berlin Heidelberg, Berlin, Heidelberg), pp. 1–41 (2008).
66. R Alexander, Solving ordinary differential equations I: nonstiff problems (E. hairer, s. p. norsett, and g. wanner). *SIAM Rev.* **32**, 485–486 (1990).
67. A El Alaoui, A Montanari, An information-theoretic view of stochastic localization. *IEEE Transactions on Inf. Theory* **68**, 7423–7426 (2022).
68. C Villani, , et al., *Optimal transport: old and new.* (Springer) Vol. 338, (2009).
